# Supplementary material for: Social network responses to victims of potentially traumatic events: A systematic review using qualitative evidence synthesis
Source: PLoS One. 2022 Nov 16;17(11):e0276476. doi: 10.1371/journal.pone.0276476 (PMC9668175; doi:10.1371/journal.pone.0276476)
Supplement: S1 Table — (DOCX) [file pone.0276476.s001.docx]

| **S1 Table. COREQ Assessment per study** |  |  |  |  |  |  |  |  |  |  |  |  |  |  |  |  |  |
| --- | --- | --- | --- | --- | --- | --- | --- | --- | --- | --- | --- | --- | --- | --- | --- | --- | --- |
|  | **46** | **47** | **48** | **49** | **50** | **51** | **52** | **53** | **54** | **34** | **55** | **56** | **57** | **58** | **59** | **60** | **61** |
| **Items** |  |  |  |  |  |  |  |  |  |  |  |  |  |  |  |  |  |
| 1. Interviewer/facilitator | x | x |  | x | x | x | x | x |  |  | x | x | x | x | x | x | x |
| 2. Credentials | x | x | x | x |  | x | x | x | x |  |  |  |  |  | x | x |  |
| 3. Occupation | x |  | x | x | x | x | x |  | x |  |  | x |  |  |  | x |  |
| 4. Gender | x |  |  | x | x |  | x |  |  | x |  |  |  |  |  | x |  |
| 5. Experience and training | x |  |  | x |  | x | x |  |  |  |  | x | x |  | x |  | x |
| 6. Relationship established |  |  |  |  |  |  |  |  |  |  |  |  | x |  | x |  |  |
| 7. Participant knowledge of the interviewer |  |  |  |  |  |  |  |  |  |  |  | x |  |  |  |  |  |
| 8. Interviewer characteristics |  | x | x | x |  | x |  | x |  |  |  |  |  |  | x | x |  |
| 9. Methodological orientation and theory | x | x | x | x | x | x | x | x | x | x | x |  |  | x | x | x | x |
| 10. Sampling | x |  | x | x |  | x | x | x |  | x | x |  | x | x | x | x | x |
| 11. Method of approach | x |  | x |  | x |  | x | x | x | x |  | x | x |  |  | x | x |
| 12. Sample size | x | x | x | x | x | x | x | x | x | x | x | x | x | x | x | x | x |
| 13. Non-participation |  |  |  |  | x | x |  | x |  |  |  |  |  |  |  | x |  |
| 14. Setting of data-collection | x |  | x | x |  | x | x | x |  | x | x |  |  |  |  | x | x |
| 15. Presence of non-participants |  |  | x |  |  |  |  | x |  | x |  |  |  |  |  |  |  |
| 16. Description of sample | x | x | x | x | x | x | x | x | x |  | x | x | x | x | x | x |  |
| 17. Interview guide | x | x | x | x | x | x | x | x | x | x | x | x | x | x | x |  | x |
| 18. Repeat interviews |  |  |  |  |  | x |  | x |  | x |  |  |  |  | x |  |  |
| 19. Audio/visual recording | x | x | x |  | x | x | x | x | x | x | x | x | x | x | x | x | x |
| 20. Field notes |  | x |  |  | x | x |  | x |  | x | x |  |  |  |  |  | x |
| 21. Duration | x | x | x |  | x | x | x |  |  | x |  | x | x |  | x | x | x |
| 22. Data saturation |  |  |  |  |  | x |  |  |  |  |  |  |  |  |  |  | x |
| 23. Transcripts returned |  |  |  |  |  | x | x |  |  |  |  |  |  |  |  |  |  |
| 24. Number of data coders | x | x | x | x |  | x | x | x |  |  | x | x | x | x | x | x | x |
| 25. Description of the coding tree |  |  |  |  | x | x |  | x |  |  |  |  |  | x | x | x |  |
| 26. Derivation of themes | x | x | x | x | x | x | x | x | x | x | x | x | x | x | x | x | x |
| 27. Software |  | x |  |  |  |  |  |  |  | x | x |  |  |  |  |  |  |
| 28. Participant checking |  |  |  |  |  |  |  |  |  | x |  |  |  |  | x |  |  |
| 29. Quotations presented | x | x | x | x | x | x | x | x | x | x | x | x | x | x | x | x | x |
| 30. Data and findings consistent | x | x | x | x | x | x | x | x | x | x | x | x | x | x | x | x | x |
| 31. Clarity of major themes | x | x | x | x | x | x | x | x | x | x | x | x | x | x | x | x | x |
| 32. Clarity of minor themes | x | x | x | x | x | x | x | x | x | x |  | x | x | x | x | x | x |
| *Comments* |  |  |  |  |  |  |  |  |  |  |  |  |  |  |  |  |  |
|  |  |  |  |  |  |  |  |  |  |  |  |  |  |  |  |  |  |
| **Total** | **20** | **17** | **19** | **18** | **18** | **25** | **21** | **22** | **13** | **19** | **15** | **16** | **16** | **14** | **21** | **21** | **18** |
| [...] refer to the reference number of the original paper | | |  |  |  |  |  |  |  |  |  |  |  |  |  |  |  |
| x in cell of the table means that the item is reported in the paper | | | |  |  |  |  |  |  |  |  |  |  |  |  |  |  |
| n/a the item is not applicable for this type of research | | |  |  |  |  |  |  |  |  |  |  |  |  |  |  |  |

| **S1 Table. COREQ Assessment per study** |  | |  | | |  | | |  | |  | | | |  |  | |  | |  | |  | |  | | | | | |  | | | | | | |  | | | | | | | | | | | |  | | | | | | | |  | | | | | |  | |  |  |  |  |  |  |  |  |  |  |  |  |  |  |  |  |  |
| --- | --- | --- | --- | --- | --- | --- | --- | --- | --- | --- | --- | --- | --- | --- | --- | --- | --- | --- | --- | --- | --- | --- | --- | --- | --- | --- | --- | --- | --- | --- | --- | --- | --- | --- | --- | --- | --- | --- | --- | --- | --- | --- | --- | --- | --- | --- | --- | --- | --- | --- | --- | --- | --- | --- | --- | --- | --- | --- | --- | --- | --- | --- | --- | --- | --- | --- | --- | --- | --- | --- | --- | --- | --- | --- | --- | --- | --- | --- | --- | --- | --- |
|  | **62** | **63** | | **64** | **65** | | **66** | **67** | | **68** | | **69** | **70** | **71** | | | **72** | | **32** | | **73** | | **74** | | **75** | | | | **76** | | | | | | **77 & 78** | | | | | | | | | |  |  |  |  |  |  |  |  |  |  |  |  |  |  |  |  |  |  |  |  |  |  |  |  |  |  |  |  |  |  |  |  |  |  |  |  |  |
| **Items** |  |  | |  |  | |  |  | |  | |  |  |  | | |  | |  | |  | |  | |  | | | |  | | | | | |  | | | | | | | | | |  |  |  |  |  |  |  |  |  |  |  |  |  |  |  |  |  |  |  |  |  |  |  |  |  |  |  |  |  |  |  |  |  |  |  |  |  |
| 1. Interviewer/facilitator | x | x | |  | x | | n/a |  | | x | | x | x | x | | | x | |  | | x | | x | | x | | | | x | | | | | |  | | | | | | | | | |  |  |  |  |  |  |  |  |  |  |  |  |  |  |  |  |  |  |  |  |  |  |  |  |  |  |  |  |  |  |  |  |  |  |  |  |  |
| 2. Credentials | x |  | | x | x | | x |  | |  | | x | x |  | | | x | |  | |  | | x | |  | | | | x | | | | | | x | | | | | | | | | |  |  |  |  |  |  |  |  |  |  |  |  |  |  |  |  |  |  |  |  |  |  |  |  |  |  |  |  |  |  |  |  |  |  |  |  |  |
| 3. Occupation | x |  | | x | x | |  |  | |  | | x | x |  | | |  | |  | |  | |  | |  | | | |  | | | | | | x | | | | | | | | | |  |  |  |  |  |  |  |  |  |  |  |  |  |  |  |  |  |  |  |  |  |  |  |  |  |  |  |  |  |  |  |  |  |  |  |  |  |
| 4. Gender | x |  | |  | x | |  |  | |  | | x |  |  | | |  | |  | | x | |  | |  | | | |  | | | | | |  | | | | | | | | | |  |  |  |  |  |  |  |  |  |  |  |  |  |  |  |  |  |  |  |  |  |  |  |  |  |  |  |  |  |  |  |  |  |  |  |  |  |
| 5. Experience and training | x |  | |  | x | | n/a |  | |  | | x |  |  | | | x | |  | | x | |  | |  | | | |  | | | | | |  | | | | | | | | | |  |  |  |  |  |  |  |  |  |  |  |  |  |  |  |  |  |  |  |  |  |  |  |  |  |  |  |  |  |  |  |  |  |  |  |  |  |
| 6. Relationship established |  |  | |  |  | | n/a |  | | x | | x |  |  | | |  | |  | |  | |  | |  | | | |  | | | | | |  | | | | | | | | | |  |  |  |  |  |  |  |  |  |  |  |  |  |  |  |  |  |  |  |  |  |  |  |  |  |  |  |  |  |  |  |  |  |  |  |  |  |
| 7. Participant knowledge of the interviewer |  |  | |  |  | | n/a |  | |  | | x |  |  | | |  | |  | |  | |  | |  | | | |  | | | | | |  | | | | | | | | | |  |  |  |  |  |  |  |  |  |  |  |  |  |  |  |  |  |  |  |  |  |  |  |  |  |  |  |  |  |  |  |  |  |  |  |  |  |
| 8. Interviewer characteristics | x | x | |  | x | | n/a |  | |  | | x |  |  | | |  | |  | |  | |  | |  | | | |  | | | | | | x | | | | | | | | | |  |  |  |  |  |  |  |  |  |  |  |  |  |  |  |  |  |  |  |  |  |  |  |  |  |  |  |  |  |  |  |  |  |  |  |  |  |
| 9. Methodological orientation and theory | x | x | | x | x | | x | x | |  | | x | x | x | | | x | | x | | x | | x | | x | | | | x | | | | | | x | | | | | | | | | |  |  |  |  |  |  |  |  |  |  |  |  |  |  |  |  |  |  |  |  |  |  |  |  |  |  |  |  |  |  |  |  |  |  |  |  |  |
| 10. Sampling |  | x | |  | x | |  | x | | x | |  | x | x | | | x | | x | |  | | x | | x | | | | x | | | | | | x | | | | | | | | | |  |  |  |  |  |  |  |  |  |  |  |  |  |  |  |  |  |  |  |  |  |  |  |  |  |  |  |  |  |  |  |  |  |  |  |  |  |
| 11. Method of approach | x | x | | x | x | | x |  | | x | |  | x |  | | |  | | x | | x | | x | | x | | | | x | | | | | | x | | | | | | | | | |  |  |  |  |  |  |  |  |  |  |  |  |  |  |  |  |  |  |  |  |  |  |  |  |  |  |  |  |  |  |  |  |  |  |  |  |  |
| 12. Sample size | x | x | | x | x | | x | x | | x | | x | x | x | | | x | | x | | x | | x | | x | | | | x | | | | | | x | | | | | | | | | |  |  |  |  |  |  |  |  |  |  |  |  |  |  |  |  |  |  |  |  |  |  |  |  |  |  |  |  |  |  |  |  |  |  |  |  |  |
| 13. Non-participation | x | x | |  | x | | x |  | |  | | x |  |  | | |  | |  | | x | |  | | x | | | | x | | | | | |  | | | | | | | | | |  |  |  |  |  |  |  |  |  |  |  |  |  |  |  |  |  |  |  |  |  |  |  |  |  |  |  |  |  |  |  |  |  |  |  |  |  |
| 14. Setting of data-collection | x | x | | x | x | | n/a | x | |  | | x | x | x | | | x | | x | | x | | x | | x | | | | x | | | | | |  | | | | | | | | | |  |  |  |  |  |  |  |  |  |  |  |  |  |  |  |  |  |  |  |  |  |  |  |  |  |  |  |  |  |  |  |  |  |  |  |  |  |
| 15. Presence of non-participants |  |  | |  |  | | n/a |  | |  | |  |  | x | | |  | |  | |  | | x | | x | | | | x | | | | | |  | | | | | | | | | |  |  |  |  |  |  |  |  |  |  |  |  |  |  |  |  |  |  |  |  |  |  |  |  |  |  |  |  |  |  |  |  |  |  |  |  |  |
| 16. Description of sample | x | x | | x | x | | x | x | | x | |  | x |  | | | x | | x | | x | | x | | x | | | | n/a? | | | | | |  | | | | | | | | | |  |  |  |  |  |  |  |  |  |  |  |  |  |  |  |  |  |  |  |  |  |  |  |  |  |  |  |  |  |  |  |  |  |  |  |  |  |
| 17. Interview guide | x | x | | x | x | | x | x | | x | | x | x | x | | | x | | x | | x | | x | | x | | | | x | | | | | | x | | | | | | | | | |  |  |  |  |  |  |  |  |  |  |  |  |  |  |  |  |  |  |  |  |  |  |  |  |  |  |  |  |  |  |  |  |  |  |  |  |  |
| 18. Repeat interviews |  |  | |  |  | | n/a |  | | x | | x |  |  | | |  | |  | | x | |  | |  | | | |  | | | | | | x | | | | | | | | | |  |  |  |  |  |  |  |  |  |  |  |  |  |  |  |  |  |  |  |  |  |  |  |  |  |  |  |  |  |  |  |  |  |  |  |  |  |
| 19. Audio/visual recording | x | x | | x | x | | n/a | x | | x | | x | x | x | | | x | |  | | x | |  | |  | | | | x | | | | | | x | | | | | | | | | |  |  |  |  |  |  |  |  |  |  |  |  |  |  |  |  |  |  |  |  |  |  |  |  |  |  |  |  |  |  |  |  |  |  |  |  |  |
| 20. Field notes | x |  | |  | x | | n/a |  | |  | |  |  | x | | |  | |  | | x | |  | |  | | | | x | | | | | | x | | | | | | | | | |  |  |  |  |  |  |  |  |  |  |  |  |  |  |  |  |  |  |  |  |  |  |  |  |  |  |  |  |  |  |  |  |  |  |  |  |  |
| 21. Duration |  | x | | x | x | | n/a | x | |  | | x | x | x | | | x | | x | | x | |  | | x | | | |  | | | | | | x | | | | | | | | | |  |  |  |  |  |  |  |  |  |  |  |  |  |  |  |  |  |  |  |  |  |  |  |  |  |  |  |  |  |  |  |  |  |  |  |  |  |
| 22. Data saturation |  |  | |  |  | |  |  | |  | |  |  | x | | | x | |  | |  | |  | |  | | | | x | | | | | |  | | | | | | | | | |  |  |  |  |  |  |  |  |  |  |  |  |  |  |  |  |  |  |  |  |  |  |  |  |  |  |  |  |  |  |  |  |  |  |  |  |  |
| 23. Transcripts returned |  |  | |  |  | | n/a |  | |  | |  | x |  | | |  | |  | |  | |  | |  | | | |  | | | | | |  | | | | | | | | | |  |  |  |  |  |  |  |  |  |  |  |  |  |  |  |  |  |  |  |  |  |  |  |  |  |  |  |  |  |  |  |  |  |  |  |  |  |
| 24. Number of data coders | x | x | | 4 | x | | x |  | |  | | x | x | x | | | x | | x | | x | | x | | x | | | |  | | | | | | x | | | | | | | | | |  |  |  |  |  |  |  |  |  |  |  |  |  |  |  |  |  |  |  |  |  |  |  |  |  |  |  |  |  |  |  |  |  |  |  |  |  |
| 25. Description of the coding tree |  |  | | 6 |  | |  |  | | x | | x |  |  | | | x | | x | |  | |  | | x | | | | x | | | | | | x | | | | | | | | | |  |  |  |  |  |  |  |  |  |  |  |  |  |  |  |  |  |  |  |  |  |  |  |  |  |  |  |  |  |  |  |  |  |  |  |  |  |
| 26. Derivation of themes | x | x | | x | x | | x | x | |  | | x | x | x | | | x | | x | | x | | x | | x | | | | x | | | | | | x | | | | | | | | | |  |  |  |  |  |  |  |  |  |  |  |  |  |  |  |  |  |  |  |  |  |  |  |  |  |  |  |  |  |  |  |  |  |  |  |  |  |
| 27. Software |  | x | |  | x | | x |  | | x | |  |  | x | | | x | |  | | x | |  | |  | | | | x | | | | | | x | | | | | | | | | |  |  |  |  |  |  |  |  |  |  |  |  |  |  |  |  |  |  |  |  |  |  |  |  |  |  |  |  |  |  |  |  |  |  |  |  |  |
| 28. Participant checking |  |  | |  |  | | n/a |  | |  | |  |  |  | | |  | |  | |  | |  | |  | | | |  | | | | | | x | | | | | | | | | |  |  |  |  |  |  |  |  |  |  |  |  |  |  |  |  |  |  |  |  |  |  |  |  |  |  |  |  |  |  |  |  |  |  |  |  |  |
| 29. Quotations presented | x | x | | x | x | | x | x | | x | | x | x | x | | | x | | x | | x | | x | | x | | | | x | | | | | | x | | | | | | | | | |  |  |  |  |  |  |  |  |  |  |  |  |  |  |  |  |  |  |  |  |  |  |  |  |  |  |  |  |  |  |  |  |  |  |  |  |  |
| 30. Data and findings consistent | x | x | | x | x | | x | x | | x | | x | x | x | | | x | | x | | x | | x | | x | | | | x | | | | | | x | | | | | | | | | |  |  |  |  |  |  |  |  |  |  |  |  |  |  |  |  |  |  |  |  |  |  |  |  |  |  |  |  |  |  |  |  |  |  |  |  |  |
| 31. Clarity of major themes | x | x | | x | x | | x | x | | x | | x | x | x | | | x | | x | | x | | x | | x | | | | x | | | | | | x | | | | | | | | | |  |  |  |  |  |  |  |  |  |  |  |  |  |  |  |  |  |  |  |  |  |  |  |  |  |  |  |  |  |  |  |  |  |  |  |  |  |
| 32. Clarity of minor themes | x | x | | x | x | | x | x | | x | | x | x | x | | | x | | x | | x | | x | | x | | | | x | | | | | | x | | | | | | | | | |  |  |  |  |  |  |  |  |  |  |  |  |  |  |  |  |  |  |  |  |  |  |  |  |  |  |  |  |  |  |  |  |  |  |  |  |  |
| *Comments* |  |  | |  |  | | *survey* |  | |  | |  |  |  | | |  | |  | |  | |  | |  | | | |  | | | | | |  | | | | | | | | | |  |  |  |  |  |  |  |  |  |  |  |  |  |  |  |  |  |  |  |  |  |  |  |  |  |  |  |  |  |  |  |  |  |  |  |  |  |
|  |  |  | |  |  | |  |  | |  | |  |  |  | | |  | |  | |  | |  | |  | | | |  | | | | | |  | | | | | | | | | |  |  |  |  |  |  |  |  |  |  |  |  |  |  |  |  |  |  |  |  |  |  |  |  |  |  |  |  |  |  |  |  |  |  |  |  |  |
| **Total** | **21** | **19** | | **17** | **24** | | **14** | **13** | | **15** | | **23** | **19** | **18** | | | **20** | | **15** | | **21** | | **16** | | **18** | | | | **21** | | | | | | **21** | | | | | | | | | |  |  |  |  |  |  |  |  |  |  |  |  |  |  |  |  |  |  |  |  |  |  |  |  |  |  |  |  |  |  |  |  |  |  |  |  |  |
| [...] refer to the reference number of the original paper | | | | | | | | | | | | | | | | | | | | | | | | | | | | | | | | | | | | | | | | | | | | | | | | | | | | | | | | | | | | | | | | | |  |  |  |  |  |  |  |  |  |  |  |  |  |  |  |  |
| x in cell of the table means that the item is reported in the paper | | | | | | | | | | | | | | | | | | | | | | | | | |  |  |  | | |  |  |  |  | |  | | |  | |  | |  | | |  | |  | | |  | |  |  |  |  |  |  |  |  |  |  |  |  |  |  |  |  |  |  |  |  |  |  |  |  |  |  |  |  |  |
| n/a the item is not applicable for this type of research | | | | | | | | | | | | | | | | | | | | | | | | | | | | | | | | | | | | | |  | |  | |  | |  | | |  | | |  | |  | |  |  |  | |  |  |  |  |  | |  | | | | | | | | | | | | | | | | | |

| **S1 Table. COREQ Assessment per study** | |  | |  | | |  | |  | | |  | |  | | |  | | | | |  | | | | | | |  | | | | | | |  | | | | | |  |  |  |  |  |  |  |  |  |  |  |  |
| --- | --- | --- | --- | --- | --- | --- | --- | --- | --- | --- | --- | --- | --- | --- | --- | --- | --- | --- | --- | --- | --- | --- | --- | --- | --- | --- | --- | --- | --- | --- | --- | --- | --- | --- | --- | --- | --- | --- | --- | --- | --- | --- | --- | --- | --- | --- | --- | --- | --- | --- | --- | --- | --- |
|  | | **79** | | **80** | | | **81** | | **82** | | | **83** | | **84** | | | **85** | | | | | **86** | | | | | | | **87** | | | | | | | **88** | | | | | |  |  |  |  |  |  |  |  |  |  |  |  |
| **Items** | |  | |  | | |  | |  | | |  | |  | | |  | | | | |  | | | | | | |  | | | | | | |  | | | | | |  |  |  |  |  |  |  |  |  |  |  |  |
| 1. Interviewer/facilitator | | x | | x | | | n/a | | x | | | x | |  | | | x | | | | |  | | | | | | | n/a | | | | | | | n/a | | | | | |  |  |  |  |  |  |  |  |  |  |  |  |
| 2. Credentials | |  | |  | | |  | | x | | |  | |  | | | x | | | | |  | | | | | | |  | | | | | | | x | | | | | |  |  |  |  |  |  |  |  |  |  |  |  |
| 3. Occupation | |  | |  | | |  | |  | | |  | |  | | | x | | | | |  | | | | | | | x | | | | | | | x | | | | | |  |  |  |  |  |  |  |  |  |  |  |  |
| 4. Gender | | x | |  | | |  | |  | | |  | | x | | | x | | | | |  | | | | | | | x | | | | | | | x | | | | | |  |  |  |  |  |  |  |  |  |  |  |  |
| 5. Experience and training | | x | | x | | | n/a | |  | | | x | |  | | |  | | | | | x | | | | | | | n/a | | | | | | | n/a | | | | | |  |  |  |  |  |  |  |  |  |  |  |  |
| 6. Relationship established | |  | |  | | | n/a | |  | | |  | |  | | |  | | | | |  | | | | | | | n/a | | | | | | | n/a | | | | | |  |  |  |  |  |  |  |  |  |  |  |  |
| 7. Participant knowledge of the interviewer | |  | |  | | | n/a | |  | | |  | |  | | |  | | | | |  | | | | | | | n/a | | | | | | | n/a | | | | | |  |  |  |  |  |  |  |  |  |  |  |  |
| 8. Interviewer characteristics | |  | |  | | | n/a | |  | | |  | |  | | | x | | | | |  | | | | | | | n/a | | | | | | | n/a | | | | | |  |  |  |  |  |  |  |  |  |  |  |  |
| 9. Methodological orientation and theory | | x | | x | | |  | | x | | | x | | x | | | x | | | | | x | | | | | | | x | | | | | | | x | | | | | |  |  |  |  |  |  |  |  |  |  |  |  |
| 10. Sampling | | x | | x | | |  | | x | | |  | | x | | | x | | | | | x | | | | | | | x | | | | | | |  | | | | | |  |  |  |  |  |  |  |  |  |  |  |  |
| 11. Method of approach | | x | |  | | | x | | x | | | x | | x | | | x | | | | |  | | | | | | | x | | | | | | | x | | | | | |  |  |  |  |  |  |  |  |  |  |  |  |
| 12. Sample size | | x | | x | | | x | | x | | | x | | x | | | x | | | | | x | | | | | | | x | | | | | | | x | | | | | |  |  |  |  |  |  |  |  |  |  |  |  |
| 13. Non-participation | |  | |  | | | x | |  | | |  | | x | | |  | | | | |  | | | | | | |  | | | | | | | x | | | | | |  |  |  |  |  |  |  |  |  |  |  |  |
| 14. Setting of data-collection | |  | | x | | | n/a | | x | | |  | | x | | |  | | | | | x | | | | | | | n/a | | | | | | | n/a | | | | | |  |  |  |  |  |  |  |  |  |  |  |  |
| 15. Presence of non-participants | |  | |  | | | n/a | |  | | |  | |  | | |  | | | | |  | | | | | | | n/a | | | | | | | n/a | | | | | |  |  |  |  |  |  |  |  |  |  |  |  |
| 16. Description of sample | | x | | x | | | x | | x | | | x | | x | | | x | | | | | x | | | | | | | x | | | | | | | x | | | | | |  |  |  |  |  |  |  |  |  |  |  |  |
| 17. Interview guide | | x | | x | | | x | |  | | | x | | x | | | x | | | | | x | | | | | | | x | | | | | | | x | | | | | |  |  |  |  |  |  |  |  |  |  |  |  |
| 18. Repeat interviews | |  | |  | | | n/a | |  | | |  | |  | | |  | | | | |  | | | | | | | n/a | | | | | | | n/a | | | | | |  |  |  |  |  |  |  |  |  |  |  |  |
| 19. Audio/visual recording | | x | | x | | | n/a | | x | | | x | | x | | | x | | | | | x | | | | | | | n/a | | | | | | | n/a | | | | | |  |  |  |  |  |  |  |  |  |  |  |  |
| 20. Field notes | |  | |  | | | n/a | |  | | | x | |  | | | x | | | | |  | | | | | | | n/a | | | | | | | n/a | | | | | |  |  |  |  |  |  |  |  |  |  |  |  |
| 21. Duration | | x | | x | | | n/a | |  | | |  | | x | | | x | | | | | x | | | | | | | n/a | | | | | | | n/a | | | | | |  |  |  |  |  |  |  |  |  |  |  |  |
| 22. Data saturation | |  | |  | | |  | | x | | | x | |  | | |  | | | | |  | | | | | | |  | | | | | | |  | | | | | |  |  |  |  |  |  |  |  |  |  |  |  |
| 23. Transcripts returned | |  | |  | | | n/a | |  | | |  | |  | | |  | | | | |  | | | | | | | n/a | | | | | | | n/a | | | | | |  |  |  |  |  |  |  |  |  |  |  |  |
| 24. Number of data coders | | x | | x | | | x | |  | | | x | | x | | | x | | | | |  | | | | | | | x | | | | | | | x | | | | | |  |  |  |  |  |  |  |  |  |  |  |  |
| 25. Description of the coding tree | |  | | x | | | x | |  | | |  | | x | | |  | | | | | x | | | | | | |  | | | | | | |  | | | | | |  |  |  |  |  |  |  |  |  |  |  |  |
| 26. Derivation of themes | | x | |  | | | x | |  | | | x | | x | | | x | | | | | x | | | | | | | x | | | | | | | x | | | | | |  |  |  |  |  |  |  |  |  |  |  |  |
| 27. Software | |  | | x | | |  | |  | | |  | | x | | | x | | | | | x | | | | | | | x | | | | | | |  | | | | | |  |  |  |  |  |  |  |  |  |  |  |  |
| 28. Participant checking | |  | |  | | | n/a | |  | | |  | | x | | |  | | | | |  | | | | | | | n/a | | | | | | | n/a | | | | | |  |  |  |  |  |  |  |  |  |  |  |  |
| 29. Quotations presented | | x | | x | | | x | | x | | | x | | x | | | x | | | | | x | | | | | | | x | | | | | | | x | | | | | |  |  |  |  |  |  |  |  |  |  |  |  |
| 30. Data and findings consistent | | x | | x | | | x | | x | | | x | | x | | | x | | | | | x | | | | | | | x | | | | | | | x | | | | | |  |  |  |  |  |  |  |  |  |  |  |  |
| 31. Clarity of major themes | | x | | x | | | x | | x | | | x | | x | | | x | | | | | x | | | | | | | x | | | | | | | x | | | | | |  |  |  |  |  |  |  |  |  |  |  |  |
| 32. Clarity of minor themes | | x | | x | | | x | | x | | | x | | x | | | x | | | | | x | | | | | | | x | | | | | | | x | | | | | |  |  |  |  |  |  |  |  |  |  |  |  |
| *Comments* | |  | | **complete study methods reported elsewhere* | | | *Survey* | |  | | |  | |  | | | **reference to Peter-Hagene & Ullman 2016 for study description* | | | | |  | | | | | | | *survey* | | | | | | | *survey* | | | | | |  |  |  |  |  |  |  |  |  |  |  |  |
| **Total** | | **17** | | **17** | | | **12** | | **14** | | | **16** | | **20** | | | **21** | | | | | **16** | | | | | | | **15** | | | | | | | **15** | | | | | |  |  |  |  |  |  |  |  |  |  |  |  |
| [...] refer to the reference number of the original paper | | | | | | | | | | | | | | | | | | | | | | | | | | | | | | | | | | | | | | | | | |  | |  | |  | |  |  |  |  |  |  |
| x in cell of the table means that the item is reported in the paper | | | | | | | | | | | | | | | | | | | | | | | | | | | | | | | | | | | | |  |  |  |  |  | |  | |  | |  |  |  |  |  |  |  |
| n/a the item is not applicable for this type of research | | | | | | | | | | | | | | | | | | | | | | | | | | | | | | | | | | | | | | | | | |  | |  | |  | |  |  |  |  |  |  |
| **S1 Table. COREQ Assessment per study** |  | |  | |  |  | |  | |  |  | |  | |  |  | |  |  |  |  | |  |  |  |  |  |  |  |  |  |  |  |  |  |  |  |  |  |  |  |  |  |  |  |  |  |  |  |  |  |  |  |
|  | **33** | | **89** | | **90** | **91** | | **92** | | **93** | **94** | | **95** | | **35** | **96** | | **97** | **98** | **99** | **100** | |  |  |  |  |  |  |  |  |  |  |  |  |  |  |  |  |  |  |  |  |  |  |  |  |  |  |  |  |  |  |  |
| **Items** |  | |  | |  |  | |  | |  |  | |  | |  |  | |  |  |  |  | |  |  |  |  |  |  |  |  |  |  |  |  |  |  |  |  |  |  |  |  |  |  |  |  |  |  |  |  |  |  |  |
| 1. Interviewer/facilitator | x | |  | | x |  | |  | |  | x | |  | |  | x | |  | x |  | x | |  |  |  |  |  |  |  |  |  |  |  |  |  |  |  |  |  |  |  |  |  |  |  |  |  |  |  |  |  |  |  |
| 2. Credentials | x | |  | |  | x | |  | | x |  | |  | | x |  | | x | x | x |  | |  |  |  |  |  |  |  |  |  |  |  |  |  |  |  |  |  |  |  |  |  |  |  |  |  |  |  |  |  |  |  |
| 3. Occupation | x | |  | |  | x | |  | | x |  | |  | | x | x | | x | x | x |  | |  |  |  |  |  |  |  |  |  |  |  |  |  |  |  |  |  |  |  |  |  |  |  |  |  |  |  |  |  |  |  |
| 4. Gender |  | |  | |  |  | |  | |  |  | |  | |  | x | |  |  |  |  | |  |  |  |  |  |  |  |  |  |  |  |  |  |  |  |  |  |  |  |  |  |  |  |  |  |  |  |  |  |  |  |
| 5. Experience and training | x | |  | |  |  | |  | |  | x | |  | |  | x | |  |  |  |  | |  |  |  |  |  |  |  |  |  |  |  |  |  |  |  |  |  |  |  |  |  |  |  |  |  |  |  |  |  |  |  |
| 6. Relationship established |  | |  | |  |  | |  | |  |  | |  | |  |  | | x | x |  |  | |  |  |  |  |  |  |  |  |  |  |  |  |  |  |  |  |  |  |  |  |  |  |  |  |  |  |  |  |  |  |  |
| 7. Participant knowledge of the interviewer |  | |  | |  |  | |  | |  |  | |  | |  |  | | x |  |  |  | |  |  |  |  |  |  |  |  |  |  |  |  |  |  |  |  |  |  |  |  |  |  |  |  |  |  |  |  |  |  |  |
| 8. Interviewer characteristics | x | |  | |  |  | |  | |  |  | |  | |  |  | |  |  |  |  | |  |  |  |  |  |  |  |  |  |  |  |  |  |  |  |  |  |  |  |  |  |  |  |  |  |  |  |  |  |  |  |
| 9. Methodological orientation and theory | x | | x | | x | x | | x | | x | x | | x | | x |  | | x | x | x | x | |  |  |  |  |  |  |  |  |  |  |  |  |  |  |  |  |  |  |  |  |  |  |  |  |  |  |  |  |  |  |  |
| 10. Sampling | x | | x | | x | x | | x | | x |  | | x | |  | x | | x |  | x | x | |  |  |  |  |  |  |  |  |  |  |  |  |  |  |  |  |  |  |  |  |  |  |  |  |  |  |  |  |  |  |  |
| 11. Method of approach | x | | x | | x |  | | x | | x | x | | x | | x |  | | x | x | x | x | |  |  |  |  |  |  |  |  |  |  |  |  |  |  |  |  |  |  |  |  |  |  |  |  |  |  |  |  |  |  |  |
| 12. Sample size | x | | x | | x | x | | x | | x | x | | x | | x | x | | x | x | x | x | |  |  |  |  |  |  |  |  |  |  |  |  |  |  |  |  |  |  |  |  |  |  |  |  |  |  |  |  |  |  |  |
| 13. Non-participation |  | |  | |  | x | |  | |  |  | |  | |  |  | | x |  |  |  | |  |  |  |  |  |  |  |  |  |  |  |  |  |  |  |  |  |  |  |  |  |  |  |  |  |  |  |  |  |  |  |
| 14. Setting of data-collection | x | |  | | x |  | |  | |  |  | | x | | x | x | | x | x | x | x | |  |  |  |  |  |  |  |  |  |  |  |  |  |  |  |  |  |  |  |  |  |  |  |  |  |  |  |  |  |  |  |
| 15. Presence of non-participants |  | |  | |  |  | |  | |  |  | |  | |  |  | |  |  |  |  | |  |  |  |  |  |  |  |  |  |  |  |  |  |  |  |  |  |  |  |  |  |  |  |  |  |  |  |  |  |  |  |
| 16. Description of sample | x | | x | | x |  | | x | |  | x | |  | | x | x | | x | x | x | x | |  |  |  |  |  |  |  |  |  |  |  |  |  |  |  |  |  |  |  |  |  |  |  |  |  |  |  |  |  |  |  |
| 17. Interview guide | x | | x | | x |  | | x | |  | x | | x | | x | x | | x | x | x | x | |  |  |  |  |  |  |  |  |  |  |  |  |  |  |  |  |  |  |  |  |  |  |  |  |  |  |  |  |  |  |  |
| 18. Repeat interviews | x | |  | |  |  | | x | |  | x | | x | |  | x | |  | x |  |  | |  |  |  |  |  |  |  |  |  |  |  |  |  |  |  |  |  |  |  |  |  |  |  |  |  |  |  |  |  |  |  |
| 19. Audio/visual recording | x | | x | | x | x | | x | |  |  | | x | | x | x | | x | x | x | x | |  |  |  |  |  |  |  |  |  |  |  |  |  |  |  |  |  |  |  |  |  |  |  |  |  |  |  |  |  |  |  |
| 20. Field notes | x | |  | |  | x | | x | |  |  | |  | | x |  | |  | x |  | x | |  |  |  |  |  |  |  |  |  |  |  |  |  |  |  |  |  |  |  |  |  |  |  |  |  |  |  |  |  |  |  |
| 21. Duration | x | |  | | x | x | | x | |  | x | | x | | x | x | | x | x | x | x | |  |  |  |  |  |  |  |  |  |  |  |  |  |  |  |  |  |  |  |  |  |  |  |  |  |  |  |  |  |  |  |
| 22. Data saturation | x | | x | | x | x | | x | |  |  | |  | |  |  | |  | x |  | x | |  |  |  |  |  |  |  |  |  |  |  |  |  |  |  |  |  |  |  |  |  |  |  |  |  |  |  |  |  |  |  |
| 23. Transcripts returned |  | |  | |  | x | |  | |  |  | |  | |  |  | |  |  |  |  | |  |  |  |  |  |  |  |  |  |  |  |  |  |  |  |  |  |  |  |  |  |  |  |  |  |  |  |  |  |  |  |
| 24. Number of data coders | x | | x | | x | x | |  | |  |  | |  | | x |  | |  | x | x | x | |  |  |  |  |  |  |  |  |  |  |  |  |  |  |  |  |  |  |  |  |  |  |  |  |  |  |  |  |  |  |  |
| 25. Description of the coding tree |  | |  | |  |  | | x | |  | x | |  | | x |  | |  |  |  | x | |  |  |  |  |  |  |  |  |  |  |  |  |  |  |  |  |  |  |  |  |  |  |  |  |  |  |  |  |  |  |  |
| 26. Derivation of themes |  | | x | | x |  | | x | | x | x | | x | | x |  | | x | x | x | x | |  |  |  |  |  |  |  |  |  |  |  |  |  |  |  |  |  |  |  |  |  |  |  |  |  |  |  |  |  |  |  |
| 27. Software | x | | x | | x |  | | x | |  | x | |  | |  |  | | x | x |  | x | |  |  |  |  |  |  |  |  |  |  |  |  |  |  |  |  |  |  |  |  |  |  |  |  |  |  |  |  |  |  |  |
| 28. Participant checking |  | |  | |  | x | |  | | x |  | |  | |  |  | |  |  |  |  | |  |  |  |  |  |  |  |  |  |  |  |  |  |  |  |  |  |  |  |  |  |  |  |  |  |  |  |  |  |  |  |
| 29. Quotations presented | x | | x | | x | x | | x | | x | x | | x | | x | x | | x | x | x | x | |  |  |  |  |  |  |  |  |  |  |  |  |  |  |  |  |  |  |  |  |  |  |  |  |  |  |  |  |  |  |  |
| 30. Data and findings consistent | x | | x | | x | x | | x | | x | x | | x | | x | x | | x | x | x | x | |  |  |  |  |  |  |  |  |  |  |  |  |  |  |  |  |  |  |  |  |  |  |  |  |  |  |  |  |  |  |  |
| 31. Clarity of major themes | x | | x | | x | x | | x | | x | x | | x | | x | x | | x | x | x | x | |  |  |  |  |  |  |  |  |  |  |  |  |  |  |  |  |  |  |  |  |  |  |  |  |  |  |  |  |  |  |  |
| 32. Clarity of minor themes | x | | x | |  | x | | x | |  | x | | x | | x | x | | x | x | x | x | |  |  |  |  |  |  |  |  |  |  |  |  |  |  |  |  |  |  |  |  |  |  |  |  |  |  |  |  |  |  |  |
| *Comments* |  | |  | |  |  | |  | |  |  | |  | |  |  | |  |  |  |  | |  |  |  |  |  |  |  |  |  |  |  |  |  |  |  |  |  |  |  |  |  |  |  |  |  |  |  |  |  |  |  |
|  |  | |  | |  |  | |  | |  |  | |  | |  |  | |  |  |  |  | |  |  |  |  |  |  |  |  |  |  |  |  |  |  |  |  |  |  |  |  |  |  |  |  |  |  |  |  |  |  |  |
| **Total** | **23** | | **15** | | **17** | **17** | | **18** | | **11** | **16** | | **14** | | **18** | **16** | | **20** | **22** | **17** | **20** | |  |  |  |  |  |  |  |  |  |  |  |  |  |  |  |  |  |  |  |  |  |  |  |  |  |  |  |  |  |  |  |
| [...] refer to the reference number of the original paper | | | | | | | | | | | | | | | | | | | | | | |  |  |  |  |  |  |  |  |  |  |  |  |  |  |  |  |  |  |  |  |  |  |  |  |  |  |  |  |  |  |  |
| x in cell of the table means that the item is reported in the paper | | | | | | | | | | | | | | | | | | | | | | | | |  |  |  |  |  |  |  |  |  |  |  |  |  |  |  |  |  |  |  |  |  |  |  |  |  |  |  |  |  |
| n/a the item is not applicable for this type of research | | | | | | | | | | | | | | | | | | | | | | |  |  |  |  |  |  |  |  |  |  |  |  |  |  |  |  |  |  |  |  |  |  |  |  |  |  |  |  |  |  |  |

| **S1 Table. COREQ Assessment per study** |  |  |  |  |  |  |  |  |  |  |  |  |  |  |  |  |  |  |  |
| --- | --- | --- | --- | --- | --- | --- | --- | --- | --- | --- | --- | --- | --- | --- | --- | --- | --- | --- | --- |
|  | **101** | **102** | **103** | **104** | **105** | **106** | **107** | **108** | **109** | **110** | **111** | **112** | **113** | **114** | **115** | **116** |  |  |  |
| **Items** |  |  |  |  |  |  |  |  |  |  |  |  |  |  |  |  |  |  |  |
| 1. Interviewer/facilitator | x | x | x |  |  | x |  | x | x | x |  | x | x |  | x | x |  |  |  |
| 2. Credentials | x | x | x | x | x | x | x | x | x | x | x | x |  |  | x |  |  |  |  |
| 3. Occupation | x | x | x |  | x | x | x | x | x | x | x | x |  | x | x | x |  |  |  |
| 4. Gender | x | x | x |  |  | x |  | x | x |  |  | x |  |  | x | x |  |  |  |
| 5. Experience and training | x | x | x |  |  |  |  | x |  |  | x | x |  |  | x |  |  |  |  |
| 6. Relationship established | x |  |  |  |  |  |  |  |  |  |  |  |  |  |  | x |  |  |  |
| 7. Participant knowledge of the interviewer |  |  |  |  |  |  |  |  |  |  |  |  |  |  |  |  |  |  |  |
| 8. Interviewer characteristics | x |  | x |  |  |  |  | x | x |  |  | x |  |  | x |  |  |  |  |
| 9. Methodological orientation and theory | x |  |  | x | x | x | x | x | x | x | x | x | x | x | x | x |  |  |  |
| 10. Sampling |  | x | x |  | x | x | x | x | x | x | x | x |  | x | x |  |  |  |  |
| 11. Method of approach | x | x | x | x | x | x |  | x | x | x | x | x |  | x | x |  |  |  |  |
| 12. Sample size | x | x | x | x | x | x | x | x | x | x | x | x | x | x | x | x |  |  |  |
| 13. Non-participation |  |  |  |  |  |  |  |  |  |  |  |  |  |  |  | x |  |  |  |
| 14. Setting of data-collection | x | x | x | x |  |  | x | x |  |  |  |  | x |  |  |  |  |  |  |
| 15. Presence of non-participants |  |  |  |  |  |  |  |  |  |  |  |  |  |  |  |  |  |  |  |
| 16. Description of sample | x | x |  | x | x | x | x | x | x | x | x | x | x | x | x | x |  |  |  |
| 17. Interview guide | x | x | x | x | x | x | x | x | x | x | x | x | x | x |  | x |  |  |  |
| 18. Repeat interviews |  |  |  |  | x | x | x |  | x |  |  |  |  |  |  |  |  |  |  |
| 19. Audio/visual recording | x | x | x | x | x | x | x | x | x | x | x | x | x | x | x | x |  |  |  |
| 20. Field notes |  |  |  |  |  |  | x |  | x | x | x |  |  |  |  |  |  |  |  |
| 21. Duration | x | x | x | x |  | x | x |  | x | x | x |  | x | x |  |  |  |  |  |
| 22. Data saturation |  |  |  |  |  |  | x |  |  | x |  | x | x |  | x |  |  |  |  |
| 23. Transcripts returned | x |  |  |  |  |  |  |  |  |  |  |  |  |  |  |  |  |  |  |
| 24. Number of data coders | x | x | x |  | x | x | x |  | x | x | x | x | x |  | x | x |  |  |  |
| 25. Description of the coding tree |  | x |  |  |  |  |  |  |  |  |  |  |  |  |  |  |  |  |  |
| 26. Derivation of themes | x | x | x | x |  | x | x | x | x | x | x | x | x | x |  | x |  |  |  |
| 27. Software |  | x | x | x |  | x |  |  | x | x |  | x |  |  | x |  |  |  |  |
| 28. Participant checking |  |  |  |  |  |  |  |  | x | x |  |  |  |  |  |  |  |  |  |
| 29. Quotations presented | x | x | x | x | x | x | x | x | x | x | x | x | x | x | x | x |  |  |  |
| 30. Data and findings consistent | x | x | x | x | x | x | x | x | x | x | x | x | x | x | x | x |  |  |  |
| 31. Clarity of major themes | x | x | x | x | x | x | x | x | x | x | x | x | x | x | x | x |  |  |  |
| 32. Clarity of minor themes | x | x | x | x | x | x | x | x | x | x | x | x | x | x | x | x |  |  |  |
| *Comments* |  |  |  |  | **description of method and results larger study presented elsewhere* |  |  |  |  |  |  |  |  |  |  |  |  |  |  |
| **Total** | **22** | **21** | **20** | **15** | **15** | **20** | **19** | **19** | **23** | **21** | **18** | **21** | **15** | **14** | **19** | **16** |  |  |  |
| [...] refer to the reference number of the original paper | | | | | | | | | | | | | | | | |  |  |  |
| x in cell of the table means that the item is reported in the paper | | | | | | | | | | | | | | | | | | |  |
| n/a the item is not applicable for this type of research | | | | | | | | | | | | | | | | |  |  |  |
