# Supplementary material for: Social network responses to victims of potentially traumatic events: A systematic review using qualitative evidence synthesis
Source: PLoS One. 2022 Nov 16;17(11):e0276476. doi: 10.1371/journal.pone.0276476 (PMC9668175; doi:10.1371/journal.pone.0276476)
Supplement: S2 Table — (DOCX) [file pone.0276476.s002.docx]

**S2 Table. Victims' experiences with social support from different support providers in the informal network with references to the included studies.**

|  | PTE and publication number | | | | |
| --- | --- | --- | --- | --- | --- |
|  | Accidents | Disasters | Homicide | Intimate Partner Violence | Sexual offences |
| **TYPE OF SUPPORTIVE RESPONSE** | | | | | |
| **Practical help (such as food, goods, and money)** | | | | | |
| -Family | [33,92] | [34,55-57,65,67,70-72] |  | [97,100-102,104-106,109,114] | [74,75,81] |
| -Friends | [91,93] | [55,65,67,70,72] | [50] | [96,97,100-102,104,106,114] | [32,74,75,81] |
| -Work/school colleagues | [33] | [55,57] |  |  | [75] |
| -Neighbors | [92,93] | [55-57,68,71,72] |  |  |  |
| -Fellow victims |  |  |  | [114] |  |
| -Religious group members |  | [34,55,65] |  |  |  |
| -Community | [93] | [34,55-57,68-70] |  | [106] |  |
| -Offender's family |  |  |  | [109] |  |
| -Social network in general | [33,91-93] | [34,55-58,65,67-72] | [50] | [96,97,100-102,104-107,109,114] | [32,74,75,81,83] |
| **Information (regarding the trauma, grief, practical issues etc.)** | | | | | |
| -Family |  | [70] |  | [109] | [81] |
| -Friends |  | [57,70] |  | [109] | [32,81] |
| -Neighbors |  | [57] |  |  |  |
| -Fellow victims |  |  |  | [109,114] |  |
| -Religious group members |  |  |  |  | [82] |
| -Community |  |  |  | [100,109] |  |
| -Social network in general | [90] | [57,70] | [53] | [100,107,109,114] | [32,81-83] |
| **Emotional help** | | | | | |
| -Family | [33,92] | [55,57,61,62,65,69,70,72] |  | [96,97,100,101,105,106,109,114] | [74,81,83,85] |
| -Friends |  | [55,57,61,65,69,70,72] | [53] | [96,97,100,101,105,114] | [32,74,75,81,83,85] |
| -Work/school colleagues |  | [55,69] |  |  |  |
| -Neighbors |  | [55,57,71,72] |  |  |  |
| -Fellow victims |  | [34,57,61] |  | [105] |  |
| -Religious group members |  | [55,61] |  | [105] |  |
| -Community |  | [55-57,68,70] |  | [106] | [75,84] |
| -Social network in general | [33,92] | [34,55-58,61,62,65,68-72] | [53] | [35,96,97,100,101,105,106,109,114] | [32,74,75,81,83-85,87] |
| **Advice (advice and guidance)** | | | | | |
| -Family |  |  | [53] | [100,102] | [80] |
| -Friends |  |  | [53] |  | [80] |
| -Neighbors |  |  |  |  | [80] |
| -Community |  | [55] |  | [106,107] | [80] |
| -Social network in general |  | [55] | [53] | [99,100,102,106,107] | [80,88] |
| **Companionship (company, not being alone)** | | | | | |
| -Family |  | [59,64,66,71] | [50,52] |  |  |
| -Friends |  | [64] |  |  |  |
| -Fellow victims |  | [64] |  |  |  |
| -Community |  | [55] |  |  | [77] |
| -Social network in general | [33] | [55,59,64,66,71] | [50,52] |  | [77] |
| **Empathy (warmth, sympathy, compassion and understanding)** | | | | | |
| -Family |  | [59,67] | [50] |  | [32,75,82] |
| -Friends |  | [67] |  | [107] | [32] |
| -Neighbors |  |  |  | [96] |  |
| -Fellow victims |  |  | [46,47,50,54] |  |  |
| -Religious group members |  |  |  |  | [82] |
| -Community |  |  | [50] | [107] |  |
| -Social network in general | [33] | [59,60,67,72] | [46,47,50,54] | [96,107] | [32,75,82,84,86-88] |
| **(Attempt to) intervene (confronting perpetrator)** | | | | | |
| -Family |  |  |  | [104,109,111,114] |  |
| -Friends |  |  |  | [104] |  |
| -Community |  |  |  | [112] |  |
| -Offender's family |  |  |  | [109,114] |  |
| -Social network in general |  |  |  | [104,107,109,111,112,114] |  |
| **Listening ear (opportunity to tell and retell stories, listening, taking time)** | | | | | |
| -Family |  | [55,57,59,62,72] |  |  | [81] |
| -Friends |  | [55,57,72] | [50] | [106] | [81,88] |
| -Neighbors |  | [56] |  |  |  |
| -Fellow victims |  | [34] | [50] |  |  |
| -Religious group members |  | [55] |  |  | [82] |
| -Community |  | [56,64,68] |  | [106,107] |  |
| -Social network in general |  | [34,55-57,59,62,64,68,72] | [48,50] | [35,106,107] | [81,82,84,86,88] |
| **Mobilizing support (encouragement help-seeking, mobilizing support, referral)** | | | | | |
| -Family |  |  |  | [96,97,100,103,109] | [32,75,85] |
| -Friends |  |  |  | [96,97,100,106,115] | [32,75] |
| -Work/school colleagues |  |  |  | [115] |  |
| -Neighbors |  |  |  | [97] |  |
| -Social network in general |  |  |  | [96,97,100,103,106,109,115] | [32,75,84-86] |
| **No unsupportive responses** | | | | | |
| -Family |  |  |  | [106] | [74,81] |
| -Friends |  |  |  |  | [74,81] |
| -Social network in general |  |  |  | [106] | [74,81,83,84] |
| **Respect autonomy (not telling what to do)** | | | | | |
| -Family |  |  |  |  |  |
| -Friends |  |  | [50] | [107] |  |
| -Community |  |  |  | [107] |  |
| -Social network in general |  |  | [50] | [107] |  |
| **Safety (safe environment to vent feelings)** | | | | | |
| -Family |  |  |  |  | [73] |
| -Friends |  |  | [53] |  |  |
| -Fellow victims |  |  | [47,50] | [109] |  |
| -Social network in general |  |  | [47,50,53] | [109] | [73] |
| **Seeking Justice (seeking justice and revenge)** | | | | | |
| -Family |  | [69] |  |  | [32,75] |
| -Friends |  | [69] |  |  |  |
| -Work/school colleagues |  | [69] |  |  |  |
| -Community |  |  |  |  | [77] |
| -Social network in general |  | [69] |  |  | [32,75,77] |
| **Sharing experiences (sharing experiences with people with the same or other traumatic event)** | | | | | |
| -Family |  | [55] | [52,54] |  | [74,81] |
| -Friends |  | [55] |  |  | [74,75,81,84,87] |
| -Fellow victims | [90] | [34,55-57,62-64,66,69] | [46,48,54] | [110,114] | [87] |
| -Religious group members |  | [55] |  |  |  |
| -Social network in general | [90] | [34,55-57,62-64,66,69] | [46,48,52,54] | [110,114] | [74,75,81,84,87] |
| **Solidarity (solidarity, community cohesion)** | | | | | |
| -Family |  | [58] | [47,50,52] |  |  |
| -Fellow victims |  | [69] |  | [114] |  |
| -Community |  | [55-58,66-70] |  |  |  |
| -Social network in general |  | [55-58,66-70] | [47,50,52] | [114] | [87] |
| **Unconditional support (unconditional, not subject to any special terms or conditions)** | | | | | |
| -Family | [33] |  | [50,51] | [101] |  |
| -Friends | [89] |  |  | [101] |  |
| -Social network in general | [33,89] |  | [50,51] | [101] |  |
| **Validation (acknowledging feelings/incident)** | | | | | |
| -Family |  |  |  | [109,111] | [32,74,75,81] |
| -Friends |  |  |  |  | [32,74,75,81] |
| -Work/school colleagues |  |  |  |  | [75] |
| -Community |  |  |  | [105] |  |
| -Social network in general |  |  |  | [105,109,111] | [32,74,75,81,84-88] |
| **TYPE OF INSUFFICIENT RESPONSE** | | | | | |
| **Insufficient practical help** | | | | | |
| -Family | [92] |  |  | [99] | [81] |
| -Friends |  |  |  | [99] | [81] |
| -Social network in general | [92] | [60,61] |  | [99] | [81] |
| **Insufficient emotional help** | | | | | |
| -Family | [93] | [34,69] | [46] | [99,102] | [81] |
| -Friends | [93] |  |  |  | [81,83] |
| -Work/school colleagues |  |  |  | [106] |  |
| -Fellow victims |  | [64,65] | [46] |  |  |
| -Community |  | [34] |  |  |  |
| -Social network in general | [33,92,93] | [34,58,64,65,69] | [46] | [99,102,106] | [75,81,83] |
| **TYPE OF UNSUPPORTIVE RESPONSE** | | | | | |
| **Abandonment (abandoning, disappearing)** | | | | | |
| -Family |  | [60,61,69] | [46,47,49] | [101] | [75,80,81] |
| -Friends | [33,89] | [60] | [47,48] | [101] | [85] |
| -Work/school colleagues |  | [61,69] |  |  |  |
| -Religious group members |  | [61] |  |  | [82] |
| -Community |  | [61,69] |  |  | [84] |
| -Social network in general | [33,89] | [60,61,69] | [46-49] | [101] | [75,80-82,84,85] |
| **Avoidance (ignoring incident or victim)** | | | | | |
| -Family |  | [61,69] | [46,54] | [102] | [81] |
| -Friends | [93] |  | [46,49] | [104,110] |  |
| -Community |  |  |  | [104] |  |
| -Offenders family |  |  |  | [109] | [74] |
| -Social network in general | [93] | [61,69] | [46,49,54] | [102,104,109,110] | [74,81] |
| **Blaming (blaming or judging responses)** | | | | | |
| -Family | [92] |  |  | [99,101,102,106,108,113] | [32,73-76,79,81-83,86] |
| -Friends |  |  |  | [99,102,106] | [32,73,74,79,81,82,84,85] |
| -Work/school colleagues |  |  | [46] |  | [76] |
| -Religious group members |  |  |  | [104] | [79,82] |
| -Community |  |  |  | [104,110] | [84] |
| -Offenders family |  |  |  | [98,108,109] |  |
| -Social network in general | [92,93] |  | [46,49] | [98,99,101,102,104,106-110,113,114] | [32,73-76,79,81-87] |
| **Complicating responses (responses or actions which are adding burden or violence)** | | | | | |
| -Family |  |  |  | [98] | [80,85] |
| -Friends |  |  |  | [98] | [81,85] |
| -Work/school colleagues |  | [61] | [48] |  |  |
| -Neighbors |  |  |  | [107] |  |
| -Community |  | [57,61] |  | [102,107] |  |
| -Offenders family |  |  |  | [98,100,108,109] | [79] |
| -Social network in general |  | [57,61] | [48] | [98,100,102,107-109] | [79-81,85,86] |
| **Egocentric responses (support providers are reacting to emotional themselves, reacting extremely)** | | | | | |
| -Family |  | [58,63] | [46,48,49,54] |  | [74,75,77,81,85-87] |
| -Friends |  |  |  |  | [32,74,75,79,81,85,87] |
| -Religious group members |  |  |  |  | [32] |
| -Offenders family |  |  |  | [103] |  |
| -Social network in general |  | [58,63] | [46,48,49,54] | [103] | [32,74,75,77,79,81,83,85-87] |
| **Feeling pressured to move on (time frame on grieving)** | | | | | |
| -Family |  | [63] | [49,54] |  | [75,76] |
| -Friends |  | [65] | [49] |  | [75] |
| -Religious group members |  |  | [47] |  |  |
| -Social network | [93] | [63,65,69] | [46,47,49,54] |  | [75,76] |
| **Justification (justification/normalization of violence, taking offenders side)** | | | | | |
| -Family |  |  |  | [96,98,102,104,106,108,116] |  |
| -Friends |  |  |  | [96,102,106] |  |
| -Religious group members |  |  |  | [96,103,104] |  |
| -Community |  |  |  | [114] | [84] |
| -Offenders family |  |  |  | [103,108,116] |  |
| -Social network in general |  |  |  | [96,98,102-104,106,108,114,116] | [84] |
| **Minimizing (minimizing incident, doubting, disbelief)** | | | | | |
| -Family |  |  |  | [110,111,113,114,116] | [74,75,79,82,85] |
| -Friends |  |  |  | [111-114] | [32,74,75,82,84] |
| -Work/school colleagues |  |  |  | [114] | [32] |
| -Community |  |  |  | [110,113] | [75] |
| -Social network in general |  |  |  | [35,107,110-114,116] | [32,74,75,79,82,84-87] |
| **Lack of empathy (insensitive, lacking in understanding, no compassion, unsympathetic)** | | | | | |
| -Family | [33,92] | [69] |  | [99,102] | [76,87] |
| -Friends | [92,93] | [69] |  | [99,102] | [87] |
| -Work/school colleagues |  | [69] | [48] |  | [32,87] |
| -Neighbors |  |  |  |  | [76] |
| -Religious group members |  |  |  |  | [79] |
| -Community | [89] | [69] | [54] |  | 81 [76] |
| -Social network in general | [33,89,92,93] | [62,69] | [47,48,54] | [99,102] | [32,76,77,79] |
| **No attempt to intervene (not wanting to intervene)** | | | | | |
| -Family |  |  |  | [96,102,104,106,114] |  |
| -Friends |  |  |  | [96,106,114] |  |
| -Neighbors |  |  |  | [96] |  |
| -Social network in general |  |  |  | [35,96,102,104,106,114] | [32] |
| **Not respecting autonomy (taking control, pushing, telling what to do)** | | | | | |
| -Family | [33] |  |  |  | [74,75,77,81,85,86] |
| -Friends | [33] |  |  | [104] | [74,81] |
| -Work/school colleagues |  | [65] |  |  |  |
| -Community |  |  |  | [114] |  |
| -Social network in general | [33] | [65] |  | [104,114] | [74,75,77,81,83,85,86,88] |
| **Treat different (changed behaviors)** | | | | | |
| -Family |  |  | [49] |  | [74,75,81] |
| -Friends |  |  |  |  | [74,81] |
| -Community |  |  | [49] |  |  |
| -Social network in general |  |  | [49] |  | [74,75,81,83] |
| **ABSENCE OF RESPONSE** | | | | | |
| **No support (no reaction or actively refusing help)** | | | | | |
| -Family |  | [70] | [46,47,49] | [101,104,105,113] | [75,79] |
| -Friends |  |  | [46,47,49] | [101,113] | [32,79] |
| -Religious group members |  |  |  |  | [32,79] |
| -Community | [89] |  | [49] |  |  |
| -Offenders family |  |  |  | [98] |  |
| -Social network in general | [89,92] | [70] | [46,47,49] | [98,101,104,105,113] | [32,75,79] |
