# Supplementary material for: Social network responses to victims of potentially traumatic events: A systematic review using qualitative evidence synthesis
Source: PLoS One. 2022 Nov 16;17(11):e0276476. doi: 10.1371/journal.pone.0276476 (PMC9668175; doi:10.1371/journal.pone.0276476)
Supplement: Attachment — Submitted filename: Response to Reviewers.docx [file pone.0276476.s004.docx]

Below we have described in detail how we responded to each comment. We start with the comments of reviewer 1.

**REVIEWER 1**

1. Thank you for the opportunity to review this manuscript. The paper is a qualitative evidence synthesis of social support following traumatic events. This topic should be of interest to a large audience of researchers and practitioners. Such a paper is also timely, as a reconceptualization of social support is emerging within the traumatic stress field.
   Unfortunately, I think the authors should revise their search strategy, and I do not recommend publishing the results based on the current search. According to the Prisma guidelines, it is important to conduct the search in such a way that it could be replicated. In this paper, the screening that eliminated more than 7000 papers is not described sufficiently. This procedure may also have led to bias in the selected papers, but this is hard to judge. Additionally, the search is in my opinion too inclusive, as for example a combination of eyewitness + theft + citizen + qualitative results in inclusion. Additionally, if ‘traumatic event’ is the target concept, the authors should revise their search words according to DSM-V.

**Response**

1.a. We would like to thank the reviewer for the kind words about the manuscript. Although we tried to be as transparent as possible about the exclusion of articles, we have found a few gaps in the reconstruction of this process. We have added the exclusion criteria more specifically in the methods section of the manuscript, namely: 1.) Studies not aimed at the general adult population, such as studies among rescue workers, soldiers and veterans. 2.) Studies with combined samples of victims and offenders or formal support providers, 3.) Studies focus on children and 4.) Studies of which the main focus was not on victims’ experiences of social support from their informal network (for example, studies on specific interventions or with a primary focus on alcohol/substance).

1.b. We modified the figure according to the PRISMA guidelines.

1.c. Third, the reviewer remarks that our search may have been too inclusive. We tried to formulate an optimal search strategy using very broad terms such as “trauma” and “social support” but also more specific terms such as “eyewitness” and “burglary”. This to prevent studies with slightly different terms from not emerging in our search. Our target concept was the experience of support after a potential traumatic event. These could be various potentially traumatic events such as disasters, accidents and various types of crime, although there were also a number of traumatic events that we were not interested in (see also remark 1.a.). Of course, all hits were screened and scrutinized whether they fitted our inclusion criteria and could help answer our review question.

1.d. The perception of support after experiencing a potential traumatic event was the target of the manuscript. We have chosen the word trauma because of the changing criteria of PTSD in the DSM and ICD. We have explained this additionally in the manuscript. We also used the word trauma to prevent studies with slightly different terms from not emerging in the search.

2. I have added a few additional comments that perhaps may be helpful for the authors.
   The Introduction is very short, particularly in light of the other sections, which are lengthy. Only one theory is mentioned, and the presentation of empirical findings could better reflect the current standing in the field (e.g. mention the more complicated relationship between mental health and social support after trauma that has been revealed in more recent studies). The introduction would be improved if the authors gave a rationale for why they chose to review qualitative papers and why they excluded childhood trauma and work-related trauma (which includes overlooking the extensive literature on military veterans). Additionally, it could be clearer to the reader why the focus is on type of social support providers and why they expect the processes to differ between exposure groups. The introduction could also profit from definitions of important terms, such as ‘social support’,
   ‘responses of the social network’, and ‘qualitative evidence synthesis’.

**Response**

2.a. First of all, we would like to thank the reviewer for the comments. We understood the comment and therefore made adjustments to the introduction. We have taken notice of the literature on mental health and social support. The relationship between the two, as the reviewer mentions, is complex. We further explained this in the introduction. We also explained other matters in more detail (see comments below).

2.b. In the introduction we further explained why we have chosen to examine qualitative studies. We wanted to understand the differences and similarities in specific types of support from different support providers following different types of potentially traumatic events as this is very relevant for targeted interventions to improve support to victims. Most of the existing quantitative research strongly focuses on generic forms of support and does not always consider the role of different support providers. Existing qualitative research does examine more specific forms of support and the role of different support providers, but often focuses on one type of trauma. Because these studies often focus on one type of trauma, the method of Qualitative Evidence Synthesis allows us to summarize these studies and examine similarities and differences across traumas.

2.c. We have chosen to exclude studies on children and work-related trauma. We decided to exclude studies on children because there is a lot of existing qualitative literature on children (especially childhood sexual abuse) and the synthesis would become too extensive. We excluded studies on work-related traumatic events because, due to the nature of their work, they might be more likely to be confronted with potential traumatic events compared to the general adult population. In addition, there may be work-related (preventive) interventions that may change the need for support from the (informal) network. In the introduction of the manuscript, we emphasized the focus on adults and in the methods section, we explained more clearly why we excluded work-related traumas.

2.d. We have focused on different types of support providers because previous research suggests that the experience of social support may vary depending on the support provider that provides a response. We explained this further in the introduction. Also, because many qualitative studies focus on one type of trauma, insight into the extent to which social support experiences are different for different groups is scarce. One can argue that, because the various potential traumatic events are very different, victims have different needs for support and the social network reacts differently to them.

2.e. We thank the reviewer for the comment. We added the missing definitions of social support. The definition of qualitive evidence synthesis was mentioned in the introduction.

3. Methods: Perhaps the authors would consider the prisma guidelines? The exclusion of papers need to be described better to increase transparency and ability to replicate.
   It was also not clear to me why the authors entered the papers full text in Nvivo. Were all
   parts of the papers used in the analysis?

**Response**

3.a. We consulted the Prisma guidelines already during the research process, but unfortunately did not write down the exclusion process as it should have been done. We have adapted the methods section according to the guidelines.

3.b. We entered the fulltexts in Nvivo because with the use of PDFs it is not possible to import only parts of the articles. We are well aware that in qualitative studies, authors are often confused where to put the findings. As a result, they can often be found in the conclusion or discussion section. Therefore, with the full texts being uploaded in NVivo, we were able to search for all the findings that were of use for our review question. This was very helpful. We have clarified this in the methods.

4. Tables: Is ‘social network’ an overarching term or mutually exclusive from the other columns? ‘Peers’: It would be better to clarify in the tables that peers refers to individuals sharing the same/similar trauma.

**Response**

4.a. The ‘social network’ was coded when a victim reported a response without mentioning a specific support provider. This was explained in the methods section. We have also added this to the description of the table (in the results section).

4.b. Here we have also added that by peers we are referring to individuals who have had the same or similar potential traumatic experience.

5. Results: I would consider removing all quotes. Quotes are often used in qualitative papers but are perhaps not informative in a synthesis. It was also very confusing with the different levels in the quotes, as some quotes are from single participants, while other quotes seem to be from a researcher’s conclusion/discussion?

**Response**

5. We understood the reviewer’s comment that the quotes may not have been informative enough and that the level in the quotes was difficult to distinguish. Considering this, we decided to remove the quotes. The quotes could be removed because in the review we were interested in the authors' findings and the quotes are the primary material of these findings. Sometimes we modified the text of the results slightly after removing the quotes.

6. The authors underline that the data consists of participants’ subjective perceptions. Nevertheless, throughout the manuscript, the authors use a language that implies ‘positive effects’ (e.g. “…important to deal with…”, “…have the effect of inducing or enhancing stress…”). This should be revised

**Response**

6. Because the focus of this study was on the experiences of victims, certain terminologies may be used that reflect how the victims experienced it. If they indicated that a certain response made it easier for them to cope with stress, we formulated it accordingly. Following the reviewer’s comments, we reviewed our interpretation of the findings and where necessary, replaced this language with more neutral language.

7. In limitations, please underline the difference between subjective experiences and consequences for health, relationships and other outcomes. Subjective positive experiences, although important, is not evidence that such experiences are health promoting

**Response**

7. We understand the reviewer's comments. However, this study is about the subjective experiences of victims. It is about what victims themselves say about what is supportive and unsupportive after experiencing a potential traumatic event. So, if victims indicated that they feel less stressed by supportive responses, that is a finding, regardless of whether it actually occurred or not. We decided to emphasize in the limitations that this research is about the experiences of victims.

8. Very minor point: ‘Revictimization’ is used wrongly, it is not synonymous with distressing.

**Response**

8. This was not intended and where necessary it has been adapted.

**REVIEWER 2**

I really appreciate your work. The manuscript was well written and it contributes to better understand the nature of the informal and formal support to victims. Moreover, it gives new insights in the victims need and in the response that are provided by the social and the community network. It also offers support to revise the type of support that - at the moment - is offered by government (in terms of policies adopted to support the victims, for example).
Has you stated, a limit in this work was the selected manuscript, written in English. As a consequence, manuscripts from the Western countries are those involved in your study.
I appreciate the analysis of qualitative studies. Researchers usually use quantitative method or mixed-method. However, the qualitative method offers the opportunity to better understand the participant's point of view and the opportunity to reflect on the practices adopted and their outcome.
Thank you for the opportunity to revise this work.

**Response**

We would like to thank the reviewer for the kind words about the manuscript.

**REVIEWER 3**

1. Thank you for the opportunity to review this manuscript, entitled "Responses of the social network of victims of violence, suicides, accidents and disaster: A Qualitative Evidence Synthesis." This paper presents a systematic review of qualitative studies on social reactions to survivors of potentially-traumatic events with the goal of identifying types of responses across traumas as well as differences in types of responses as a function of responder type and trauma type. Strengths include the use of a large number of databases in the search and inclusion of a large number of papers. In addition, synthesizing the research across trauma types is a worthwhile goal. However, several concerns significantly tempered my enthusiasm for this manuscript. My concerns are next presented in order of significance.
   There has been extensive characterization of types of responses received by victims in the qualitative research. This is not reviewed, nor is the need for a review justified given the state of the literature in this area. See, for example, Dworkin, Brill, & Ullman (2019) for a review of the quantitative research in this area that discusses the types of reactions received.
   Dworkin, E. R., Brill, C. D., & Ullman, S. E. (2019). Social reactions to disclosure of interpersonal violence and psychopathology: A systematic review and meta-analysis. Clinical psychology review, 101750.

**Response**

1. First, we would like to thank the reviewer for the kind words about the manuscript. First of all, the reviewer is right that Dworkin is a missing reference in the manuscript. At the time we submitted this manuscript, this review had not yet been published. Because the study is very relevant, we added Dworkin to the manuscript. About the other concern; it is true that existing qualitative research addresses different types of responses. However, the existing research often focuses on one type of event only. We carried out this synthesis in order to compare the existing qualitative research and to explore similarities and differences between the different trauma groups. We have attempted to clarify this in the introduction.

2. The focus on qualitative studies only, rather than both quantitative and qualitative studies, should be justified. This is especially important because the three sub-questions appear to be best addressed by quantitative methods. Relatedly, the sub-questions do not seem to match the main question. The main question is much more qualitative and addresses survivors’ experiences of the responses they receive, while the sub-questions address simply what types of responses are received and from whom, not the experiences of those responses.

**Response**

2.a. Through the comments of the reviewer we felt that we could explain this even better in the introduction and have revised the introduction accordingly. We wanted to understand the differences and similarities in specific types of support from different support providers following different types of potentially traumatic events as this is very relevant for targeted interventions to improve support to victims. Most of the existing quantitative research strongly focuses on generic forms of support and does not always consider the role of different support providers. Existing qualitative research does examine more specific forms of support and the role of different support providers, but often focuses on one type of trauma. Because these studies often focus on one type of trauma, the method of Qualitative Evidence Synthesis allows us to summarize these studies and examine similarities and differences across traumas.

2.b. We can imagine that the research questions created confusion for the reviewer. The different questions distracted from where the focus of our review lies, the experiences of victims and the similarities and differences across the different victimization groups. We therefore decided to revise the research questions and merged the sub-questions into 1 qualitative main question.

3. I am concerned that the search terms used were insufficient or inappropriately specified, resulting in a failure to identify important studies.
*Were titles and abstracts only searched, or full papers?
*It does not seem like the “victim” search terms would be needed- the “type of potentially traumatic event” terms should have been sufficient. Requiring these terms to be present in the title/abstract may have limited the results.
*The harras* search term was spelled wrong (it should have been harass*)
*The term “friend,” and maybe also “supporter,” should have been included in the “social informal network” list of search terms.
*The terms “suicide,” “homicide,” “collision,” “war,” “combat,” and “pandemic” should likely have been included in the “type of potentially traumatic event” list. Suicide in particular is a notable omission because it appears in the article title.

Potentially as a result of the search terms used, there are many studies missing from the list of sexual assault studies:
Ahrens, C. E. (2006). Being silenced: The impact of negative social reactions on the disclosure of rape. Journal of Community Psychology, 38(3–4), 263–274.
Ahrens, C. E., & Aldana, E. (2012). The ties that bind: Understanding the impact of sexual assault disclosure on survivors' relationships with friends, family, and partners. Journal of Trauma & Dissociation, 13, 226–243.
Ahrens, C. E., Cabral, G., & Abeling, S. (2009). Healing or Hurtful: Sexual Assault Survivors’ Interpretations of Social Reactions From Support Providers. Psychology of Women Quarterly, 33,81–94.
Ahrens, C. E., Stansell, J., & Jennings, A. (2010). To tell or not to tell: The impact of disclosure on sexual assault survivors’ recovery. Violence and Victims, 25(5), 631–648.
Campbell, R., Ahrens, C. E., Sefl, T., Wasco, S. M., & Barnes, H. E. (2001). Social reactions to rape victims: Healing and hurtful effects on psychological and physical health outcomes. Violence and Victims, 16(3), 287–302.
Dworkin, E. R., Pittenger, S. L. S. L., & Allen, N. E. (2016). Disclosing sexual assault within social networks: A mixed-method investigation. American Journal of Community Psychology, 57(1–2), 216–228.
Filipas, H. H., & Ullman, S. E. (2001). Social reactions to sexual assault victims from various support sources. Violence and Victims, 16(6), 673–692.
Lorenz, K., Ullman, S. E., Kirkner, A., Mandala, R., Vasquez, A. L., & Sigurvinsdottir, R. (2018). Social reactions to sexual assault disclosure: A qualitative study of informal support dyads. Violence against women, 24(12), 1497-1520.
Ullman, S. E. (1996). Do social reactions to sexual assault victims vary by support provider? Violence and Victims, 11(2), 143–157.
Ullman, S. E., Lorenz, K., & Kirkner, A. (2017). Alcohol’s role in social reactions to sexual assault disclosures: A qualitative study of informal support dyads. Journal of interpersonal violence, 0886260517721172.
Ullman, S. E., Lorenz, K., Kirkner, A., & O’Callaghan, E. (2018). Postassault substance use and coping: a qualitative study of sexual assault survivors and informal support providers. Alcoholism treatment quarterly, 36, 330–353.

There were also studies missing from the interpersonal violence list, which maybe were excluded due to saturation, but this should be clarified:
Edwards, K. M., Dardis, C. M., & Gidycz, C. A. (2012). Women’s disclosure of dating violence: A mixed methodological study. Feminism & Psychology, 22, 507-517. doi:10.1177/0959353511422280
Mahlstedt, D. & Keeny, L. (1993). Female survivors of dating violence and their social
networks. Feminism & Psychology, 3(3), 319-333.
Trotter, J.L. & Allen, N.E. (2009). The good, the bad, and the ugly: Domestic violence survivors’ experiences with their informal social networks. American Journal of Community Psychology, 43(3-4), 221-231.

**Response**

3.a. First, we would like to thank the reviewer for the comments. We've noticed that we have not properly explained certain decisions and have therefore made some adjustments. We did not use any specific filters during the search in the databases. We have clarified this in the methods section.

3.b. We have indeed used both the search term victim and terms for various potential traumatic events. We also tried to use very broad terms to make sure we didn't miss any studies.

3.c. The term “harras” was indeed misspelled, we decided to remove “harras” from the search strategy in the manuscript. Because we used the broad search term “trauma”, we do not believe that we have missed studies.

3.d. The terms “friend” and “supporter” could have been added to the search strategy. However, because we used the broad term "social support", we do not expect that we missed studies.

3.e. The terms “war” and “combat” were deliberately not included in the search because these events were not in scope. “Homicide” is included in the search strategy. “Suicide” was missing because we weren't interested in it. Therefore, we deleted it from the manuscript. “Collission” and “pandemic” are indeed missing. Because of the broad word “trauma”, these studies could have surfaced. In the limitation section we explained few things more clearly.

3.f. Regarding the list of missing studies the reviewer refers to, a number of these studies were included in the synthesis, a number of studies were excluded for various reasons and some studies did not surface:

**The following studies were included in the synthesis:**

- Ahrens, C. E. (2006). Being silenced: The impact of negative social reactions on the disclosure of rape. Journal of Community Psychology, 38(3–4), 263–274.
- Ahrens, C. E., & Aldana, E. (2012). The ties that bind: Understanding the impact of sexual assault disclosure on survivors' relationships with friends, family, and partners. Journal of Trauma & Dissociation, 13, 226–243.
- Ahrens, C. E., Cabral, G., & Abeling, S. (2009). Healing or Hurtful: Sexual Assault Survivors’ Interpretations of Social Reactions From Support Providers. Psychology of Women Quarterly, 33,81–94.
- Filipas, H. H., & Ullman, S. E. (2001). Social reactions to sexual assault victims from various support sources. Violence and Victims, 16(6), 673–692.
- Lorenz, K., Ullman, S. E., Kirkner, A., Mandala, R., Vasquez, A. L., & Sigurvinsdottir, R. (2018). Social reactions to sexual assault disclosure: A qualitative study of informal support dyads. Violence against women, 24(12), 1497-1520.
- Mahlstedt, D. & Keeny, L. (1993). Female survivors of dating violence and their social
  networks. Feminism & Psychology, 3(3), 319-333.

**The following studies were excluded with reasons:**

- Ahrens, C. E., Stansell, J., & Jennings, A. (2010). To tell or not to tell: The impact of disclosure on sexual assault survivors’ recovery. Violence and Victims, 25(5), 631–648. [??]

**Response:** This study is excluded due to research design; the qualitative part cannot be analyzed properly.

- Dworkin, E. R., Pittenger, S. L. S. L., & Allen, N. E. (2016). Disclosing sexual assault within social networks: A mixed-method investigation. American Journal of Community Psychology, 57(1–2), 216–228.

**Response**: This study is excluded because the focus is on reasons to disclose or not to disclose, and it is not about responses from support providers.

- Ullman, S. E., Lorenz, K., & Kirkner, A. (2017). Alcohol’s role in social reactions to sexual assault disclosures: A qualitative study of informal support dyads. Journal of interpersonal

violence, 0886260517721172.

**Response:** This study is excluded because the primary focus is on alcohol/substance abuse.

- Ullman, S. E., Lorenz, K., Kirkner, A., & O’Callaghan, E. (2018). Postassault substance use and coping: a qualitative study of sexual assault survivors and informal support providers. Alcoholism treatment quarterly, 36, 330–353.

**Response:** This study is excluded because the primary focus is on alcohol/substance abuse.

- Trotter, J.L. & Allen, N.E. (2009). The good, the bad, and the ugly: Domestic violence survivors’ experiences with their informal social networks. American Journal of Community Psychology, 43(3-4), 221-231.

**Response:** The sample of this study included respondents under the age of 18, which is why the study was excluded.

**Three studies did not surface:**

- Ullman, S. E. (1996). Do social reactions to sexual assault victims vary by support provider? Violence and Victims, 11(2), 143–157
- Edwards, K. M., Dardis, C. M., & Gidycz, C. A. (2012). Women’s disclosure of dating violence: A mixed methodological study. Feminism & Psychology, 22, 507-517. doi:10.1177/0959353511422280
- Campbell, R., Ahrens, C. E., Sefl, T., Wasco, S. M., & Barnes, H. E. (2001). Social reactions to rape victims: Healing and hurtful effects on psychological and physical health outcomes. Violence and Victims, 16(3), 287–302.

**Response:** The studies of Ullman (1996) and Campbell et al. (2001) did not emerge from the search but would have been excluded due to the research design. The results sections mainly show quantitative results and it is not possible to analyze the qualitative results properly. The study of Edwards did not emerge from the search either. This study would probably have been included, although the study is not rich in qualitative results. In the discussion, we explain that we may have missed studies by only performing an electronic search and we highlight the study of Edwards. The study was briefly analyzed and compared with the results from the synthesis.

4. It was unclear what parts of articles were coded. Was it the full text of the results, or only quotes from participants within the text? If only quotes from participants were coded, this approach would need a compelling justification, as it seems limited.

**Response**

4. We are aware that authors in qualitative studies do not only include findings in the results, but sometimes also in the conclusion or discussion. For this reason, we have searched the entire full text for findings relevant to our review question. We have clarified this in the methods.

5. One of the key findings of qualitative research in this area is that survivors differ in what they consider to be supportive, unsupportive, harmful, and insufficient. Some studies included (e.g., the Dworkin study) explicitly address this issue, but the analysis does not reflect this type of nuance.

**Response**

5. We believe that our review shows the differences between what victims consider to be supportive and unsupportive. For one victim, a certain response may not be supportive, while another victim may find it supportive. We have tried to clearly formulate these differences in victims’ experiences in the results. We also highlight the similarities and differences in experiences between the different victim groups. The issue as mentioned by the reviewer, is also highlighted in the discussion.

6. The key findings table (Table 4) seems to include many overlapping concepts and concepts at varying levels of breadth. It would be worth considering which concepts should be grouped together versus being presented separately. For example:
*How do “social network,” “peers,” “friends,” and “community” differ from each other?
*Empathy, validation, safety, solidarity, unconditional support, and emotional help seem highly similar.
*How do unsupportive, harmful, and insufficient reactions differ?

**Response**

6. We understand the reviewer's comments, but it was the intention of the review to show the nuances in support. It is indeed true that empathy, validation, solidarity etc. are all in the spectrum of emotional support, but they are different concepts. We also wanted to show the different layers of support and support providers and explain the nuances of responses in the text.

Minor comments:
7. *The introduction should include a definition of social support generally and the different types of support under investigation.

**Response**

7. We have added the definitions of perceived and received social support to the introduction.

8. *The text should be copy-edited for grammar, as PLOS ONE does not copy-edit manuscripts, and there were a large number of errors (e.g., “Aim of the present study is to fill this gap of scientific knowledge,” “family members earthquake”)

**Response**

8. We regret that the reviewer found a large number of errors in the manuscript. We checked the manuscript again for spelling mistakes and grammar issues. Where necessary we have adjusted the manuscript.

9. *Consider changing the title to: “Social network responses to victims of potentially-traumatic events: A qualitative evidence synthesis” or something similar to better reflect the range of events included in the search

**Response**

9. We thought this was a great suggestion and changed the title, with a small adjustment.

10. *The term “disenfranchised grief” does not seem to apply well to all types of trauma, as grief is generally considered to be specific to situations involving loss. Perhaps something like “feeling pressured to move on” would be more appropriate

**Response**

10. We thank the reviewer for this suggestion and changed the term into feeling pressured to move on.

11. *“Well-intended reactions” gets at the intentions of the support provider, not the type of support offered, so it seems inappropriate here

**Response**

11. We understand the comment of the reviewer and that the formulation and explanation might be confusing. It is a very important finding from our review. The fact that victims experience certain negative reactions as positive because they think the support provider has a good intention is quite relevant. This indicates that it is important to consider the people that gave the response and the relationship they have with the victim as well. In order to clarify the finding and avoid confusion, we removed the response from the table and adjusted the text.

12. *It was quite hard to follow the one-letter indication of the type of trauma in Table 4- is there another way this could be set up?

**Response**

12. Following the reviewer's comment, we modified the table and displayed the different trauma groups in the rows. We believe that the table is now more readable.

13. *Was the same type of support from a single study double-counted in more than one support area? For example, sexual assault research tends to combine practical help and information support, but those studies seem to be counted under both areas in Table 4.

**Response**

13. Yes, it was. So, if a victim indicated that he experienced information and practical help in general, they're both in the table. We've explained this in more detail in the methods section and added it to the description of the table.
