# Supplementary material for: Social network responses to victims of potentially traumatic events: A systematic review using qualitative evidence synthesis
Source: PLoS One. 2022 Nov 16;17(11):e0276476. doi: 10.1371/journal.pone.0276476 (PMC9668175; doi:10.1371/journal.pone.0276476)
Supplement: Attachment — Submitted filename: Response to reviewers.docx [file pone.0276476.s005.docx]

Below we have described in detail how we responded to each comment. We start with the comments of reviewer 1.

**REVIEWER 1**

1. I consider the authors have accomplished the reviewer´s comments and they have provided an improved version.
Just the first figure they have attached is not very clean (please attached a figure with better resolution).

**Response**

1. We thank the reviewer for the kind words. We attached a figure with better resolution.

**REVIEWER 2**

Thank you for the opportunity to review this manuscript. This is a systematic review of qualitative studies, aiming to answer to the following research question: How do adult victims of violence, homicides, accidents and disasters experience responses of informal support providers in the aftermath of the event, and what are similarities and/or differences in experiences across various victimization groups according to qualitative studies?

Among the strengths of this study is an original and worthwhile aim (to synthetize qualitative studies on social support after trauma, with a focus on differences sources of social support) and inclusion of a large number of studies. I sincerely applaud the authors’ interest in contributing with something new in this field, and I agree that we need new development both within theoretical and methodological approaches to social support. At the time I read this manuscript, it has received feedback from two previous reviewers, and from what I can judge, this feedback has been taken seriously and has probably increased the quality of the manuscript.

**Response**

We would like to thank the reviewer for the kind words about the manuscript.

1. The introduction does not provide a clear rationale for why it is necessary to study different groups of victims together. On what theoretical or empirical grounds do you expect differences in experience of information support providers? The introduction somewhat unfocused and includes various references suggesting that “it’s complicated” rather than a clear rationale for why your study has to be done.

**Response**

1. In response to the reviewer’s comments, we felt we could better explain this in the introduction. Social support after experiencing a potentially traumatic event can be very important for victims, especially when social support matches victims' needs (otherwise it may also have harmful effects). On the one hand, we expect certain generic needs after experiencing a potentially traumatic event. On the other hand, victims can also be expected to have specific needs (about the type of support and/or a specific support provider) depending on the type of event and their circumstances. Currently, our knowledge in this regard is scarce because the current qualitative and quantitative literature is limited in scope. Current quantitative literature is typically focused on general forms of support and has less attention for different actors within the informal network and qualitative literature is typically focused on one type of traumatic event. With this study, we aim to fill this gap in scientific knowledge. This may improve victim policies and victim services, and may also inform the informal network on how they can be supportive. The introduction has been largely rewritten to clarify the above. For this reason, we refer the reviewer to the revised introduction.

2. The results are difficult to discern – there are many details and few superordinate take-home messages.

**Response**

2. Thanks to the reviewer's comments, we modified the results to better highlight the most important results. The order of the results section and the tables made this section rather unclear. Therefore, we changed the order of the results section and made it more consistent throughout. We first explain what victims' experiences were and then elaborate on the similarities and differences. In addition, we reduced or adjusted the size of the tables to improve readability. We explain the changes in the results section in more detail later in this letter.

3. The discussion should be more connected to the theory and empirical studies mentioned in the introduction

**Response**

3. We thank the reviewer for the comment. We made several major adjustments to the discussion to better align the findings with the theory and empirical studies mentioned in the introduction. Because these were major adjustments, we would like to refer the reviewer to the rewritten discussion for all adjustments. We also explain the changes in the discussion section in more detail later in this letter.

4. Abstract:
- The background lacks an explicit and substantial objective – what is the research question of this paper? “Organizing and summarizing studies on” is a little passive. What does this study aim to teach us something about?
- I would like the findings part to be more specific and superordinate.
- I did not understand this sentence: “Various responses were experienced across victimization groups while others were more trauma-specific.”
- This sentence does not provide substantial information: “The synthesis provides several directions for future research and implications for victim assistance policies.” (which directions and implications do you mean?)

**Response**

4.a. We thank the reviewer for this suggestion. We have revised the abstract and made the aim of the study more explicit:

*“A substantial number of qualitative studies examined how adult victims of potentially traumatic events (PTEs) experienced support provided by family members, friends, colleagues, and other significant others in the informal network. Importantly, the large majority of qualitative studies focused on the perceived support of victims of specific events such as sexual offences, partner violence, homicide, accidents and disasters. Although it is likely that across specific PTEs there are similarities as well as differences in experienced support from the informal network, to date no systematic review synthesized the results of qualitative studies on support from the informal network following various types of PTEs. The aim of the present systematic review is to fill this gap in the scientific knowledge, which is also highly relevant for victim services, policymakers, and the informal network.”*

4.b. We thank the reviewer for the suggestion. We adjusted the results part of the abstract:

*“Overall, four major categories of perceived social support were identified, namely, support perceived as supportive, supportive but insufficient, unsupportive, and absent from informal support providers, which included friends, family, neighbors, (if applicable) offender’s family, religious group members, work/school colleagues, fellow victims, the local community, and the social network in general. Across the PTE groups, there were similarities in experiencing positive forms of support (particularly empathy and sharing experiences with fellow victims) as well as negative forms of support (abandonment, avoidance, lack of empathy, and not experiencing support despite victim’s request for help). There were also differences across PTE groups, in particular, victims of sexual and intimate partner violence mentioned a number of other supportive (mobilizing support, no unsupportive responses) and non-supportive (e.g., justification or normalization of violence and minimizing responses) responses.”*

4.c. We removed the sentence and adjusted the results section to make it more specific (see response 4.b.)

4.d. Based on this comment we rewritten this section of the abstract:

*“By using the method of qualitative evidence synthesis, victims’ experiences of informal social support following various PTEs were examined, while simultaneously increasing size and diversity of the overall sample. The review showed that different actors within the social informal network can play an important role in providing support after victims experience violence, homicide, accidents, and disasters. However, the review revealed that the large majority of qualitative studies were aimed at victims of IPV, and only for this type of PTE was saturation achieved. This indicates that, although this synthesis identified several similarities and differences, it is still too early to draw more definitive conclusions on similarities and differences in experienced social support after various PTEs and that future qualitative studies focusing on other PTEs are much needed.”*

5. Introduction:
- The authors have chosen to base their study on Hobfoll’s conservation resource theory. Does this theory provide any input that can shed light on the research question? Please outline.
- The authors refers to several snippets of the social support literature. But I find the overall argumentation for the research question weak – do you expect that different types of support is more prevalent after some types of traumatic events – for example – people are more emotionally supportive after terrorism than after sexual abuse? Or that people offer more instrumental support after natural disasters than after sexual abuse? Or that people receive more unhelpful responses after family violence than after accidents? Please outline this link.

**Response**

5.a. We thank the reviewer for the suggestion. According to COR theory, we expect that each victim of a potentially traumatic event will need to regain some resources and that social support may be important in this process. The problems experienced by victims and the resulting needs may depend on the type of event but also on the situation of the victim. At the same time, we also expect that certain needs will be more generic and that the type of event may trigger certain responses from others. We have modified the introduction to make this explicit:

*“Given the differences between various types of PTEs and their consequences, it is likely that part of the problems victims face and their subsequent needs for social support from the informal network are specifically related to the type of PTE and the victim’s situation [10-12]. For instance, the need for temporary housing after a devasting flood will be absent after a traffic accident. Some needs, such as the need for a listening ear and social acknowledgement, may be more general and not specifically related to the type of PTE [30,31]. In addition, the type of event may trigger specific responses from the informal network regardless of the needs of victims. For example, after violence-related events the informal network may express thoughts about the role and responsibility of the victims leading up to these events, while such thoughts will be absent after disasters that have natural causes, such as hurricanes.”*

5.b. We thank the reviewer for the suggestion, and we think the questions of the reviewer are very valid. We have tried to modify the section on existing literature to make it more clear why it is important to learn more about the experiences of different groups of victims. Currently, many of the questions cannot be answered because the current quantitative and qualitative literature is limited in scope. Therefore, we conducted this qualitative review to fill this gap in research and, more importantly, so that the information can be used to improve victim assistance. We have modified the introduction to make this more clear:

*“However, research has shown that some victims lack social support or are confronted with negative support from the informal network, suggesting that there is room for improvement [20-24]. A better fit between victims’ needs for social support and the provided support is highly relevant because research has also shown that a perceived lack of support or negative support is associated with higher stress levels and PTSD symptom levels [17,20,25-27], while ongoing stress and PTSD symptom levels over the long term may erode social support levels [28,29].”*

*“Further insight into the differences and similarities in experienced social support across different types of PTEs is highly relevant for victim services, policymakers, and the informal network. This insight may help to improve the social support provided to victims by optimizing the match between needs and social support. The outcomes of both qualitative and quantitative PTE-related studies are of relevance in this perspective. However, PTE-related empirical quantitative studies on social support have predominantly focused on associations between support on the one hand and post-event mental health (especially PTSD symptomatology) on the other hand [20,28]. These studies assessed the effects of different, general types of support, such as emotional, instrumental, informational, and esteem support. Little to no attention was paid to specific types of support following specific events, or the support received from specific support providers. When victims’ needs and circumstances vary considerably, the same general forms of support may be less appropriate, and more understanding is needed of what is perceived by different types of victims as supportive and unsupportive of their informal network. Qualitative PTE-related research on social support after trauma did focus on perceived levels of support from different support providers and the different types of responses victims have received, but this research often focuses on specific groups of victims, such as victims of sexual offences, accidents, disasters, and intimate partner violence, and as a result, there remains little understanding of the experiences of different types of victims.”*

6. Methods:
- I find that the authors have described their methods in a thorough and accurate way.

**Response**

6. We thank the reviewer for the kind words.

7. Results:
- The four paragraphs based on the four topics are nicely written, but I would like some more substantial overall extractions. There are many details here, and the answer to the research question is not clearly presented.
- The tables are somewhat intimidating, at least for this reader. I would consider place table 2 in supplemental materials, and I would try to simplify table 4. It is very extensive and it is difficult to read. It looks more like an evidence gap map (https://eppi.ioe.ac.uk/cms/Default.aspx?tabid=3790 ) than a table with substantial results. So I look at it and I wonder: What can we learn from this? Perhaps this version of this table could be put in the supplemental materials as well?

**Response**

7.a. We thank the reviewer for the comment. We have tried to make this clear by moving different parts and consistently keeping the same structure. First, we explain what the victim's experience was (supportive, unsupportive, insufficient, or absent) elaborating on the following elements:

(1) what support provider the victim mentioned in the context of this experience (2) what specific responses were mentioned in this context (if applicable) (3) why the response was perceived as supportive/unsupportive/insufficient or absent?, and (4) what consequences did victims mention from the perceived support or non-support? Second, we explain the similarities and differences between the different groups, discussing first the similarities and then the differences.

7.b. We thank the reviewer for the suggestions. The tables did not always make it clear and contained a lot of information. The reviewer's suggestion helped us to modify the large table with the main findings to a shorter, simpler version. The expanded table, with all the specific references to the original studies, was included as supplementary material. We left the table of study characteristics in the article for practical reasons and did not include it as supplementary material. This table contains the references to the original studies to which the findings table is then linked as well as the references in the text. When this table is no longer included in the article it is more difficult to retrieve which finding came from which studies. We did manage to make the table a lot smaller, it is now 2 pages instead of 4 pages.

8. - I am unsure what your main findings were. After you have reminded us about your research question, please formulate 3-5 main findings that you will discuss.
- I would like to see a discussion linking your findings to the theory and empirical studies you mention in the introduction, and integrate your findings it better in the literature. For example, I find it very interesting that you found that peers was the only support provider not mentioned as unhelpful. How would you interpret this in light of the literature showing that psychological debriefing after trauma are potentially harmful? Should we arrange for people to meet and share their experiences, or should we not?
- Please also write explicitly what your contribution to the literature was. I think that several of your findings could be results from a quantitative study as well.

**Response**

8.a. We thank the reviewer for the suggestion. At the beginning of the discussion, we made clear what our main findings were:

*“Despite our broad search terms, no qualitative studies were identified that focused on the experiences of social support from the informal network of victims of human trafficking, missing persons, kidnapping, theft, burglary, robbery, medical mistakes, or fraud. In addition, the large majority of the identified studies (n=46) focused on victims of IPV and saturation was reached for this group only. This means that much of what we know is based on the experiences of these victims and that we do not yet have a complete picture of the experiences of other victims. Therefore, we can only draw preliminary conclusions about similarities and differences between victim experiences, and further research is needed. The synthesis did reveal a number of specific supportive, unsupportive, insufficient, or lack of responses, as well as a several similarities and differences among the different PTE groups. Some of these similarities and differences were not unexpected and seem to be explained by the type of event the victims experienced, while others were more salient. We also found that the included qualitative studies did not always distinguish between different support providers, as well as existing social support questionnaires, and paid little attention to the interaction between informal support providers and victims. However, the results of the synthesis suggest that it is important to consider different support providers because specific support providers seem to play a particularly positive or negative role after a victim experiences a PTE. First, the different experiences of victims and several important similarities and differences will be explained, after which the aforementioned gaps in existing research will be addressed.”*

8.b. In response to the reviewers' comments, we made several changes to the discussion. We modified the order of the discussion to make it more organized and concrete. We also linked the findings in the discussion more directly to the theory and literature in the introduction. Among the similarities and differences that emerged in the synthesis, we highlighted some key findings. For example, we further explained the importance of peer support that emerged from the synthesis. Because we have made major revisions, please refer the reviewer to the new discussion.

8.c. We have tried to clarify this by modifying the introduction and discussion in several ways. Also, we made it more explicit in the conclusion:

*“Overall, the study revealed important similarities and differences between different PTE groups and support providers, suggesting that it is important to consider different types of PTE in future research and that a distinction between informal support providers may also provide important insights. This may help to better align victims' needs and social support and may be of great importance for victim services and targeted interventions. In addition, this comprehensive systematic review of qualitative research on social support revealed several gaps in the existing literature, the most important of which is that the current knowledge from qualitative studies is based primarily on the social support experiences of victims of IPV. Future qualitative studies should investigate whether the patterns of experienced support found in this review can be confirmed.”*
