# Supplementary material for: Social network responses to victims of potentially traumatic events: A systematic review using qualitative evidence synthesis
Source: PLoS One. 2022 Nov 16;17(11):e0276476. doi: 10.1371/journal.pone.0276476 (PMC9668175; doi:10.1371/journal.pone.0276476)
Supplement: Attachment — Submitted filename: Response to reviewers.docx [file pone.0276476.s006.docx]

Below we have described in detail how we responded to each comment. We start with the comments of the academic editor and then with the comments of reviewer 2.

**ACADEMIC EDITOR**

1.In the Introduction, lines 132 0 136 (“Qualitative PTE-related research on social support…”), please include some references to the studies you are referring to here.

**Response**

1.Thanks to the editor’s comment, we included a reference for each PTE group mentioned in the sentence:

*Qualitative PTE-related research on social support after trauma did focus on perceived levels of support from different support providers and the different types of responses victims have received, but this research often focuses on specific groups of victims, such as victims of sexual offences [32], accidents [33], disasters [34], and intimate partner violence [35], and as a result, there remains little understanding of the experiences of different types of victims.*

2.In the introduction, paragraph 5 (lines 120 – 136), the authors refer to some PTE-relevant quantitative and qualitative studies that have examined social supports, but only state what they’ve focused on, rather than what they’ve found. Please provide a brief description of the main, collective findings from these studies and highlight what gaps remain.

**Response**

2.We thank the editor for the suggestion. Earlier in the introduction we briefly describe the main findings of current research on social support:

*However, research has shown that some victims lack social support or are confronted with negative support from the informal network, suggesting that there is room for improvement [20-24]. A better fit between victims’ needs for social support and the provided support is highly relevant because research has also shown that a perceived lack of support or negative support is associated with higher stress levels and PTSD symptom levels [17,20,25-27], while ongoing stress and PTSD symptom levels over the long term may erode social support levels [28,29].*

Later in the introduction, we explain the current gaps in quantitative and qualitative research and the importance of this study:

*These studies assessed the effects of different, general types of support, such as emotional, instrumental, informational, and esteem support. Little to no attention was paid to specific types of support following specific events, or the support received from specific support providers. When victims’ needs and circumstances vary considerably, the same general forms of support may be less appropriate, and more understanding is needed of what is perceived by different types of victims as supportive and unsupportive of their informal network. Qualitative PTE-related research on social support after trauma did focus on perceived levels of support from different support providers and the different types of responses victims have received, but this research often focuses on specific groups of victims, such as victims of sexual offences, accidents, disasters, and intimate partner violence, and as a result, there remains little understanding of the experiences of different types of victims.*

We hope that with this we have sufficiently answered the editor's point.

3.In the ‘study selection’ section, lines 277 – 286, please include how many people were involved in the title/abstract screening and the full-text screening, it would also be useful to include the initials of the authors who undertook the screening at each stage. If possible, please also include what level of inter-rater agreement was reached for each stage, and how disputes in decisions were resolved.

**Response**

3.We thank the editor for the suggestion. We added the following sentence in study selection section:

*Subsequently, the remaining 914 full texts were screened, and 814 studies were excluded for reasons of research design, type of research, sample or scope of the study.* ***Title/abstract and full text screening was performed by MS.***

Because the screening of titles, abstracts and fulltext was performed by MS there was no inter-rater agreement. In coding and synthesizing the articles, there was coordination with others on the research team. This is explained in the data extraction and synthesis section: *This coding process was performed per group predominantly by MS, and it was discussed till consensus with HB and FvW was reached. & After finalizing this process, MS and FvW reviewed all concepts and then revisited and refined all concepts until all data were explained and accounted for. Furthermore, all inconsistencies, contradictions, and definitions were intensively discussed by the whole research team.*

4.In the Discussion, lines 642 – 644, it is unnecessary to restate the results in terms of number of papers found (e.g., “The synthesis identified 17 specific types of supportive responses, 12 non-supportive responses, …”). Please remove this. There are a few other sections within the Discussion where the number of papers are stated, and could be removed.

**Response**

4. We thank the editor for the comments and removed the number of papers and changed the sentence:

*The qualitative evidence synthesis revealed that victims perceived the support of the informal network after experiencing a PTE as supportive, insufficient, unsupportive, or absent and mentioned several specific (un)supportive responses that characterized these experiences*

We also checked the rest of the discussion and adjusted it where necessary.

5.I would also caution the authors to be wary that they are not simply restating the Results within the Discussion, but instead are discussing what the results mean in the context of what literature has been previously published. A final review of the Discussion by the authors might reveal section where the word count could be cut down by removing the restating of results.

**Response**

5. Thanks to the editor’s comments, we have reviewed the discussion and tried to further limit the repetition of results. Since this involves multiple changes, we refer the editor to the new discussion.

6.The ‘Challenging the measures of social support’ section in the Discussion reads more as an ‘implications’ section, rather than highlighting a gap in research – so perhaps make it its won implication section. The authors use ‘Furthermore’ a lot in this section, it feels quite repetitive and impedes the flow of the discussion. Please consider removing the ‘furthermores’.

**Response**

6. We thank the editor for the suggestion. We think the section does fit nicely in its current form because the gaps in current research are linked (both implicit and explicit) to implications for further research (see also the sections selective attention within social support research and interaction between support provider and victims). We added *implications for future research* to the subtitle of the section to make this more explicit. We also removed the use of furthermore where possible.

**REVIEWER 2**

1.Abstract: The first sentence in the conclusion repeats what is already in the methods and I do not understand the second part. I would omit this sentence.

**Response**

1.We thank the reviewer for the comment. We removed the sentence.

2.Table 3: Should it be “victims of ebola virus disease” rather than just “ebola virus disease”?

**Response**

2.We thank the reviewer for the suggestion. We changed it to *victims of ebola virus disease.*

3.Table 3: It is not clear what “Duma part I – introduction a nd method section” mean.

**Response**

3.We thank the reviewer for the suggestion. We explained it in the text (… one paper is part of one study published in two papers; one paper contains the introduction and methods sections, while the other paper contains the results and discussion sections [82,83].) but to clarify it, we have also explained it in more detail in the table: *Duma part I – this paper contains the introduction and methods section of the above paper.*

4.Table 4: Please indicate what the numbers refer to in the title or note of the table (all tables should be self-explanatory).

**Response**

4.Thanks to the reviewer’s comments, we added the following note to the table:

*The number in a cell corresponds to the number of studies in which the response was mentioned*
